# L‐Aspartic Acid with Dual Functions: An Eco‐Friendly and Affordable Choice to Accelerate High Salinity Brine Utilization

**DOI:** 10.1002/advs.202408081

**Published:** 2025-01-21

**Authors:** Suning Mao, Zhen Yu, Jie Chen, Yizhe Shen, Minjie Li, Chen Sun, Xiaoqing Lin, Bin Yang, Tong Chen, Qunxing Huang, Xiaodong Li, Jianhua Yan

**Affiliations:** ^1^ State Key Laboratory of Clean Energy Utilization Institute for Thermal Power Engineering Zhejiang University Hangzhou 310058 China; ^2^ Institute of Zhejiang University – Quzhou No. 99 Zheda Road Quzhou 324000 China; ^3^ Key Laboratory of Biomass Chemical Engineering of Ministry of Education College of Chemical and Biological Engineering Zhejiang University Hangzhou 310027 China

**Keywords:** critical relative humidity, fly ash washing leachate, interfacial solar evaporation, L‐Asp, moisture‐enabled electricity generation, vaterite

## Abstract

L‐Aspartic acid (L‐Asp) poses a dual function, which can affect the evaporation and crystallization process of the high‐salinity brine by altering the physical or chemical properties of the salts. MSWI (municipal solid waste incineration) fly ash washing leachate, as a typical high‐salinity brine, is utilized here to validate this hypothesis under the simulation guidance. Since L‐Asp has stronger adsorption energy on the (110) crystal face of CaCO_3_, L‐Asp can facilitate the preferential growth of more valuable vaterite during the softening process (pretreatment before crystallization). Subsequently, the resulting solution undergoes a stable interfacial solar‐driven crystallization process under the L‐Asp, with a high salt recovery ratio of 0.14 kg m^−2^ h^−1^ within 40 h under 1 sun. Finally, to harness the “cradle to grave” full life cycle utilization of washing leachate, the extracted mixed salts are utilized for moisture‐enabled electricity generation. L‐Asp can significantly enhance this process by reducing the critical relative humidity of mixed salts, thereby exhibiting a stable open circuit potential and short‐cut current of ≈ 0.51 V and 10.6 µA within continuous 800 min. In conclusion, this work not only provides innovative approaches for upcycling high‐salinity wastewater but also explores novel applications for L‐Asp.

## Graphical abstract

L‐Aspartic acid (L‐Asp) enhances crystallization and upcycling of high‐salinity MSWI fly ash leachate. It promotes vaterite growth during softening, modulates salt creeping behavior, and improves moisture‐enabled electricity generation performance of salt mixture by lowering critical relative humidity. This innovative approach advances brine management and explores novel applications of L‐Asp in sustainable processes.

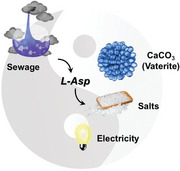

## 1 Introduction

High‐salinity brines originate from diverse industrial and chemical engineering processes, including flue gas washing effluents, coal to chemical facilities, waste incineration fly ash leachate, etc.^[^ ^1^ ^]^ Effectively managing these wastewaters to achieve zero liquid discharge (ZLD) is crucial for advancing Sustainable Development Goals but presents significant technical and energy‐related challenges, especially for brines rich in calcium ions.^[^ ^2^ ^]^ Traditional technologies always incur huge energy consumption and secondary waste (salts and precipitates) generation.^[^ ^3^ ^]^ Sequential extraction of valuable products from brine consisting of complex salts requires precise control over the precipitation and crystallization process, which remains still challenging.^[^ ^4^ ^]^

MSWI fly ash washing leachate is a typical Ca^2+^ bearing high‐salinity brine. MSWI fly ash, a hazardous byproduct generated more than 10 million tons yearly in China through waste incineration, consists of 30–45 wt.% soluble Ca‐Na‐K mixed chloride salts and insoluble aluminosilicates and gypsum.^[^ ^5^ ^]^ Based on the similar compositions between MSWI fly ash and cement‐based materials,^[^ ^6^ ^]^ in 2020, the Chinese government published a regulation to encourage recycling MSWI fly ash into cement production or cold‐bonded construction materials (HJ1134‐2020).^[^ ^7^ ^]^ However, the existence of soluble chloride salts would cause secondary pollution and severe corrosion during cement production and service life.^[^ ^8^ ^]^ Therefore, water washing has been a necessary procedure to remove and recycle chloride salts before further utilization, resulting in large amounts of high salinity brine generation.

Calcium salts in MSWI fly ash washing leachate would interfere with the salt crystallization process, resulting in scale layer formation that destroys the evaporation equipment.^[^ ^9^ ^]^ Therefore, the softening process gradually becomes a necessary pretreatment procedure before evaporation. Based on the high alkaline properties of MSWI fly ash washing leachate, the wet carbonation process would be an affordable choice for CaCO_3_ production and spontaneous brine softening.^[^ ^10^ ^]^ Finally, a traditional mechanical vapor recompression (MVR) process was used to evaporate high‐salinity leachate. During the above process, to achieve better technical and economic performance, several issues should be addressed: 1) how to control the amphoteric metals (Pb and Cd) leaching during the washing process?^[^ ^11^ ^]^ 2) How to increase the value of derived CaCO_3_ byproducts? 3) How to decrease the cost and energy consumption when evaporating the complex leachate matrix? 4) How to use the derived mixed chloride salts harvested from the crystallization process?

**Figure 1:**
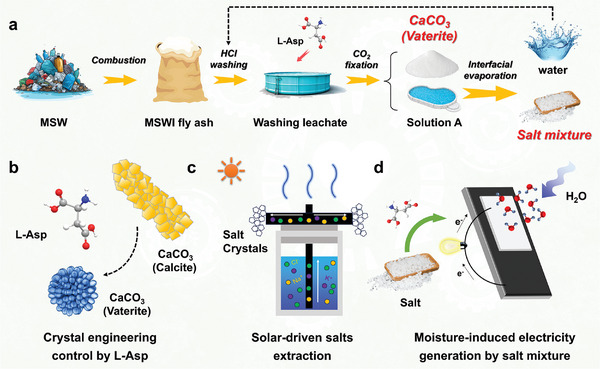
Upcycling of MSWI fly ash washing leachate to vaterite and hydroscopic salts. a) A tandem process assisted by L‐Asp to treat the MSWI fly ash washing leachate. b) CaCO_3_ crystal engineering from calcite to vaterite by L‐Asp in the softening process. c) Solar‐driven salts extraction from the high‐salinity leachate. d) Moisture‐induced electricity generation by salt mixture.

L‐Asp, as a novel scale inhibitor, could adjust the physical or chemical properties of the salts. However, to date, the effect of L‐Asp in the high‐salinity brine treatment has not been adequately explored. This highlights a gap in our understanding and emphasizes the need for further research to investigate the effect of L‐Asp. Inspired by this, a tandem process assisted by L‐Asp to treat the MSWI fly ash washing leachate was proposed (**Figure** **1**a). The production of the vaterite CaCO_3_ was regulated by L‐Asp in the softening process (Figure 1b).^[^ ^12^ ^]^ To reduce energy consumption, a solar‐driven salt extraction process was used to replace MVR to evaporate the high‐salinity leachate.^[^ ^13^ ^]^ Similarly, L‐Asp could also promote stable solar crystallization by distorting the crystal structure of the mixed inorganic salts^[^ ^14^ ^]^ (Figure 1c). As we known, it is the first report that L‐Asp can assist the solar‐driven interfacial salt extraction process. Finally, to harness the “cradle to grave” full life cycle utilization of washing leachate, the extracted mixed salts are utilized for moisture‐enabled electricity generation (Figure 1d). L‐Asp can significantly enhance this process by reducing the critical relative humidity of mixed salts, thereby exhibiting a stable electrical output and information encryption. In summary, we verified that L‐Asp poses a dual function, which could affect the evaporation and crystallization process of the high‐salinity by altering the physical or chemical properties of the salts. In conclusion, this work performed a viable recycling route for Ca^2+^ bearing high salinity brine, and elucidated the multi‐functional roles of widely available amino acid (L‐Asp) in crystallization and hydroscopic processes.

## 2 Results and Discussion

### 2.1 Dual Function of L‐Asp for Sewage Resource Utilization

**Figure 2:**
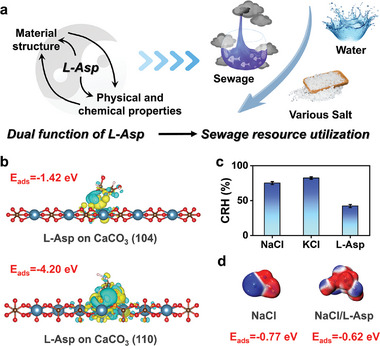
Dual function of L‐Asp in the high‐salinity brine treatment. a) Schematic diagram. b) The adsorption energy and differential charge diagram of L‐Asp on CaCO_3_ with different crystal faces. c) The critical relative humidity (CRH) of different salts. d) The adsorption energy of L‐Asp/NaCl complexes and NaCl alone to water molecule.

To achieve zero liquid discharge, it is often necessary to treat high‐salinity wastewater through evaporation and crystallization processes, finally obtaining various salt products.^[^ ^15^ ^]^ L‐Asp, a common amino acid, plays a dual function in these processes by altering the physical or chemical properties of the as‐obtained products (**Figure** **2**a). Herein, fly ash washing leachate contains various inorganic salts (calcium salts, potassium salts, sodium salts, etc.) is selected as the typical high‐salinity brine to reveal the effect of the L‐Asp.^[^ ^16^ ^]^ To prevent salt scaling, softening treatment is usually employed to eliminate calcium ions and induce the formation of inorganic precipitates such as CaCO_3_.^[^ ^17^ ^]^ L‐Asp's strong adsorption energy on the (110) crystal face over (104) of CaCO_3_ promotes the formation of valuable vaterite during the softening process (pretreatment before crystallization) (Figure 2b).^[^ ^18^ ^]^ Additionally, L‐Asp significantly alters the properties of the inorganic salts obtained through evaporation and crystallization. For instance, the critical relative humidity (the starting point for hygroscopic absorption) of NaCl and KCl exceeds 75%, whereas L‐Asp's critical relative humidity is ≈40% (Figure 2c). This result suggests that L‐Asp has the potential to lower the critical relative humidity of mixed salts. Furthermore, DFT calculation indicates that L‐Asp/NaCl complexes have lower water adsorption energies compared to NaCl alone, indicating reduced saturated moisture absorption capacity facilitated by L‐Asp (Figure 2d). These findings underscore L‐Asp's ability to substantially influence the physical and chemical characteristics of products derived from high‐salinity wastewater treatment. Subsequent sections will further elucidate the effects of L‐Asp, taking the treatment of fly ash washing leachate as an example.

### 2.2 Clean Vaterite Production through Acid Pickling and L‐Asp Modulation

To achieve clean and sufficient extraction of soluble fractions of alkaline waste MSWI fly ash, acid pickling was applied to control the leaching of Zn/Pb and the dissolution of Ca spontaneously.^[^ ^19^ ^]^ The effect of HCl dosage on the final pH value and metal leaching was shown in Figures S1–S4 (Supporting Information). The pH of water washing leachate of MSWI fly ash is ≈12. Nearly 1.5–1.8 m HCl could bring the pH of the suspensions to 9–10 at the L/S = 3 (Figure S1, Supporting Information). This indicated the high alkaline properties of raw MSWI fly ash. As shown in Figure S2 (Supporting Information), the leaching of Na and K are both unaffected by pH. Things are different for alkaline earth metals Ca and Mg, as the pH value decreased from 12 to 9, the Ca and Mg leaching increased by at least 4 times (Figure S3, Supporting Information). Further pH reduction showed little improvement on Ca extraction. As for the leaching of heavy metals which is shown in Figure S4 (Supporting Information), different metals showed distinctive characteristics as pH value changed.^[^ ^20^ ^]^ The concentrations of amphoteric metals Zn and Pb in the leachate exhibited a valley at pH 9, while most heavy metals (Cu, Cd, and Ni) remained stable and the leaching concentrations are below the national limits (Table S3, Supporting Information). Above all, through precise control on pH value, the Ca in the MSWI fly ash could be effectively dissolved in the leachate, while the toxic metals remained in the solid phase. The optimization of leaching procedure would be helpful to increase the yield and properties of CaCO_3_ by‐products.

**Figure 3:**
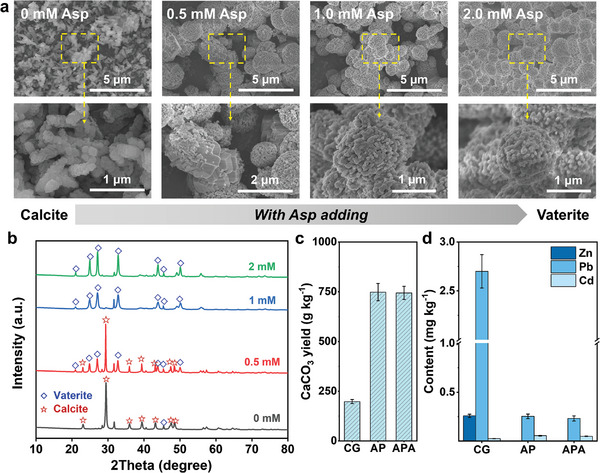
Clean and high purity vaterite production from MSWI fly ash leachate by acid pickling and L‐Asp controlled wet carbonation process. a) SEM images and b) XRD patterns of CaCO_3_ derived from wet carbonation of MSWI fly ash leachate under different dosages of L‐Asp; c) CaCO_3_ yields, d) heavy metals concentrations of CaCO_3_ from wet carbonation. All data represent the means ± SE from three independent triplicate experiments.

The first step to treat fly ash washing leachate is the softening treatment. L‐Asp was selected as a crystallization modulator to tune the morphology and facets of derived CaCO_3_. With the L‐Asp dosage increasing from 0–2 mm, the shape of CaCO_3_ changed from a long chain of cubic box to sphere‐like balls (**Figure** **3**a). The morphology change indicated a transformation from calcite to vaterite.^[^ ^21^ ^]^ XRD patterns were also applied to characterize the facet change (Figure 3b). Without L‐Asp addition, CaCO_3_ was mainly composed of calcite phase (The typical peaks were indexed to 23.06°, 29.38°, 35.96°, and 39.38°). After L‐Asp addition, the vaterite phase emerged at the CaCO_3_ (The typical peaks were indexed to 24.92°, 27.07°, 32.81°, and 43.89°).^[^ ^22^ ^]^ When the L‐Asp increased from 0.5 to 1 mM, the peaks of calcite disappeared. Further increase of L‐Asp concentration would enhance the intensity of vaterite. According to the relative intensity ratio, the purity of vaterite at L‐Asp of 2 mM reached 99.9%, suggesting a successful vaterite production with high purity (Figure S5, Supporting Information). The above results implied that L‐Asp can facilitate the preferential growth of the vaterite crystal of the CaCO_3_. The driving force (ΔG_v_) for forming stable vaterite is controlled as Equation (1) where R, T_g_, and S represent the gas constant, absolute temperature, and supersaturation, respectively.^[^ ^23^ ^]^

(1) $$\Delta G_{v}=-RT_{g}/\mathrm{nS}$$

The negatively charged carboxyl groups on the L‐Asp molecules can firmly bind to Ca^2^⁺, creating a large local supersaturated microenvironment.^[^ ^24^ ^]^ Additionally, the strong electric field generated by the high concentration of negatively charged carboxyl groups of L‐Asp facilitates interactions with the crystal planes that possess the highest positive charge. Vaterite features two calcium planes (0 0 1) and (1 0 0) with the same charge of 6.7 Ca^2+^/nm^2^, whereas calcite only poses the (0 0 1) plane with a low charge density of 4.5 Ca^2+^/nm^2^. Based on the above effects, L‐Asp can promote the preferential growth of the vaterite.^[^ ^25^ ^]^ In addition, the yields and heavy metals content of CaCO_3_ from different washing leachate were shown in Figure 3c,d. It could be easily noticed that the yield increased from 198 to 750 g kg^−1^ after HCl addition. Meanwhile, the concentrations of Zn and Pb in CaCO_3_ decreased from 0.26 and 2.7 mg kg^−1^ to 0.0015 and 0.253 mg kg^−1^, respectively. The concentration of Cd increased a little from 0.025 mg kg^−1^ to 0.055 mg kg^−1^. These results were all consistent with the leaching tests, thereby confirming the necessity of acid pickling for maximum and clean vaterite production. Moreover, L‐Asp addition showed negligible effects on CaCO_3_ yield and heavy metals behavior.

### 2.3 Solar‐Driven Salts Extraction Assisted by L‐Asp

**Figure 4:**
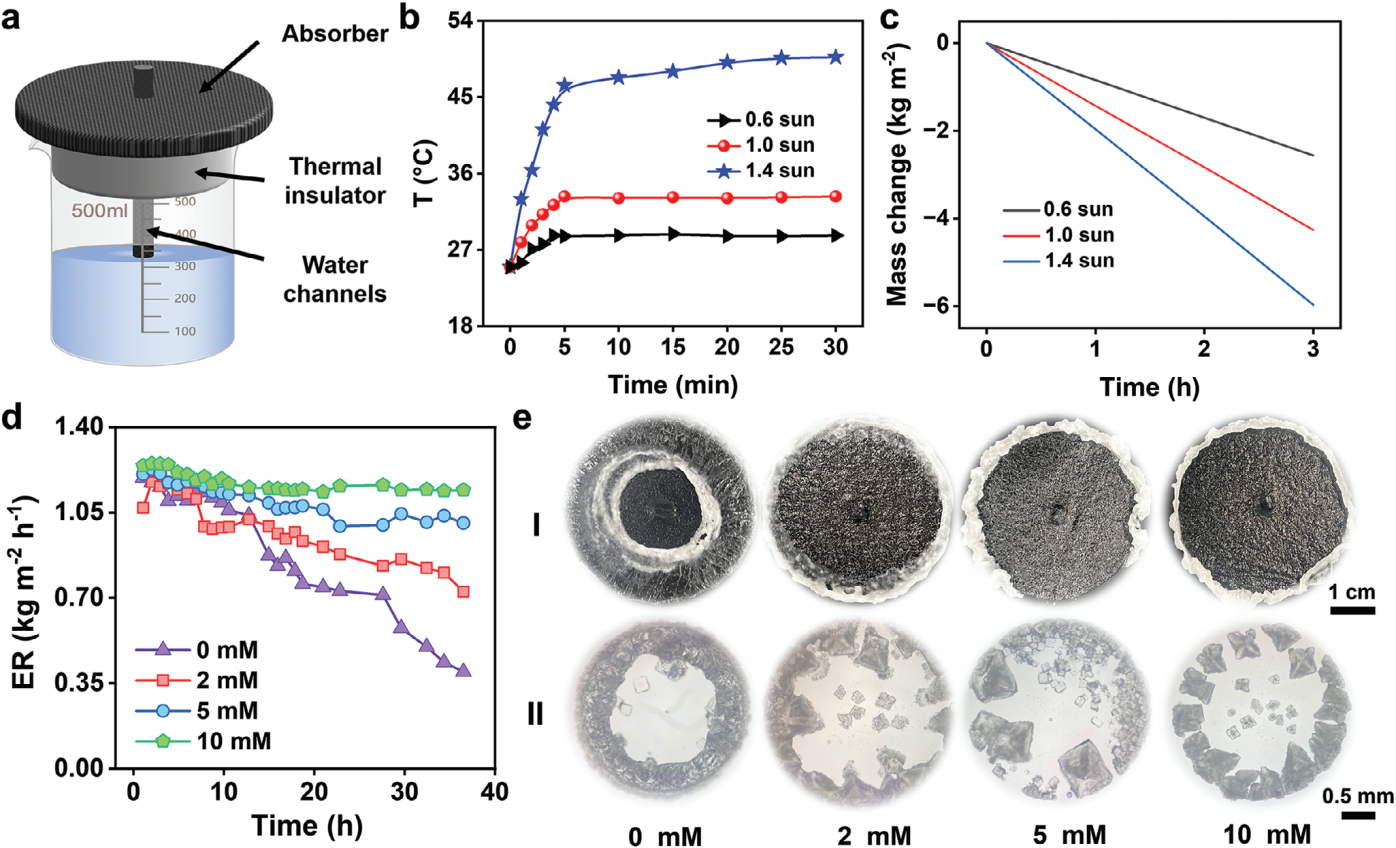
Stable solar‐driven salts harvesting assisted by L‐Asp on the CSFS. a) Schematic diagram of the CSFS; The evaporation performance of CSFS under different solar flux. b) The surface temperature varying curves and c) mass change curves as a function of time. d) The long‐term evaporation rate under different dosages of L‐Asp; e) (I) The digital photos and (II) optical images of the salt crystals generated from the fly ash washing leachate with different contents of L‐Asp.

The second step to treat fly ash washing leachate is the evaporation and crystallization process, yielding mixed salts and condensed water. L‐Asp could also assist this process. We employed the solar‐driven interfacial evaporator to process the washing leachate. The classical single‐flowing solar evaporator (CSFS, **Figure** **4**a) was used to verify this speculation.^[^ ^2^, ^26^ ^]^ The commercial carbon felt was used to fabricate the absorber and water transfer channels of the CSFS owing to its high optical absorption and hydrophilic porous structure (Figures S6 and S7, Supporting Information).^[^ ^27^ ^]^ Compared to pure water, CSFS enabled a higher evaporation rate of 1.3 kg m^−2^ h^−1^ under 1 sun (Figure S8, Supporting Information). The surface steady‐state temperature and evaporation rate of CSFS gradually rose as the solar flux increased (Figure 4b,c).

Except for high salinity, fly ash washing leachate also features a complex composition, which leads to the scaling phenomenon. As expected, CSFS failed in a stable operation in the fly ash washing leachate. Specifically, the evaporation rate of CSFS reduced from 1.2 to 0.4 kg m^−2^ h^−1^ within 36 h (Figure 4d), accompanied by the gradual appearance of salt crystals on the surface of CSFS (Figure 4e). The addition of L‐Asp could improve crystal performance. It was found that when increasing L‐Asp content, the effective evaporation diameter of CSFS also enlarged from 1.97 to 5 cm (Figure S9, Supporting Information). With the addition of 10 mM L‐Asp, the salt crystal gradually formed on the edge of the CSFS (Figure S10, Supporting Information), and the evaporation rate was maintained at 1.20 kg m^−2^ h^−1^. Note that CSFS facilitated stable evaporation of fly ash washing leachate owing to the edge preferential salt formation effect, a capability lacking in traditional evaporation structure (Figures S11 and S12, Supporting Information). The evaporation rate of the traditional evaporation structure rapidly declined to 0.6 kg m^−2^ h^−1^ within 12 h. Moreover, Moreover, the sintering dust‐washing leachate was chosen to evaluate the feasibility of this L‐Asp method on other high‐salinity wastewaters. CSFS also enabled stable evaporation and salt production under the L‐Asp dosage (Figure S13, Supporting Information), indicating that the universality of stabilization evaporation effect of L‐Asp on other brines.

Similar to distorting the crystal structure of calcium carbonate, L‐Asp also poses the ability to inorganic salts. The in situ optical microscope was used to observe the crystallization performance of CSFS in the fly ash washing leachate with different L‐Asp content (Figure 4e). Without L‐Asp addition, the salt crystals formed on CSFS were dense salt crystals, which would hinder the water transfer and vapor escape. Adding L‐Asp changes the interaction force between salt molecules, making the mixed chloride salt more porous and easier to separate.^[^ ^9^ ^]^ As the concentration of Asp increases, dense salt crystals transform from 3D chimneys to 2D rings. Meanwhile, the salt crystals gradually separate and no longer stick together, ensuring a stable evaporation rate. Therefore, L‐Asp can change the morphology and pore structure of the salt crystals, thereby affecting the evaporation performance in the crystallization process. Except for the morphology and pore structure, the crystalline phase of the salt may also affect the evaporation performance but needs further studied in the future. Finally, this method also enabled a low salt extraction cost of $ 0.0270 g^−1^ salts (Details in Supplementary Method).

### 2.4 Enhanced Moisture‐Induced Electricity Generation of L‐Asp Modified Salt Mixture

**Figure 5:**
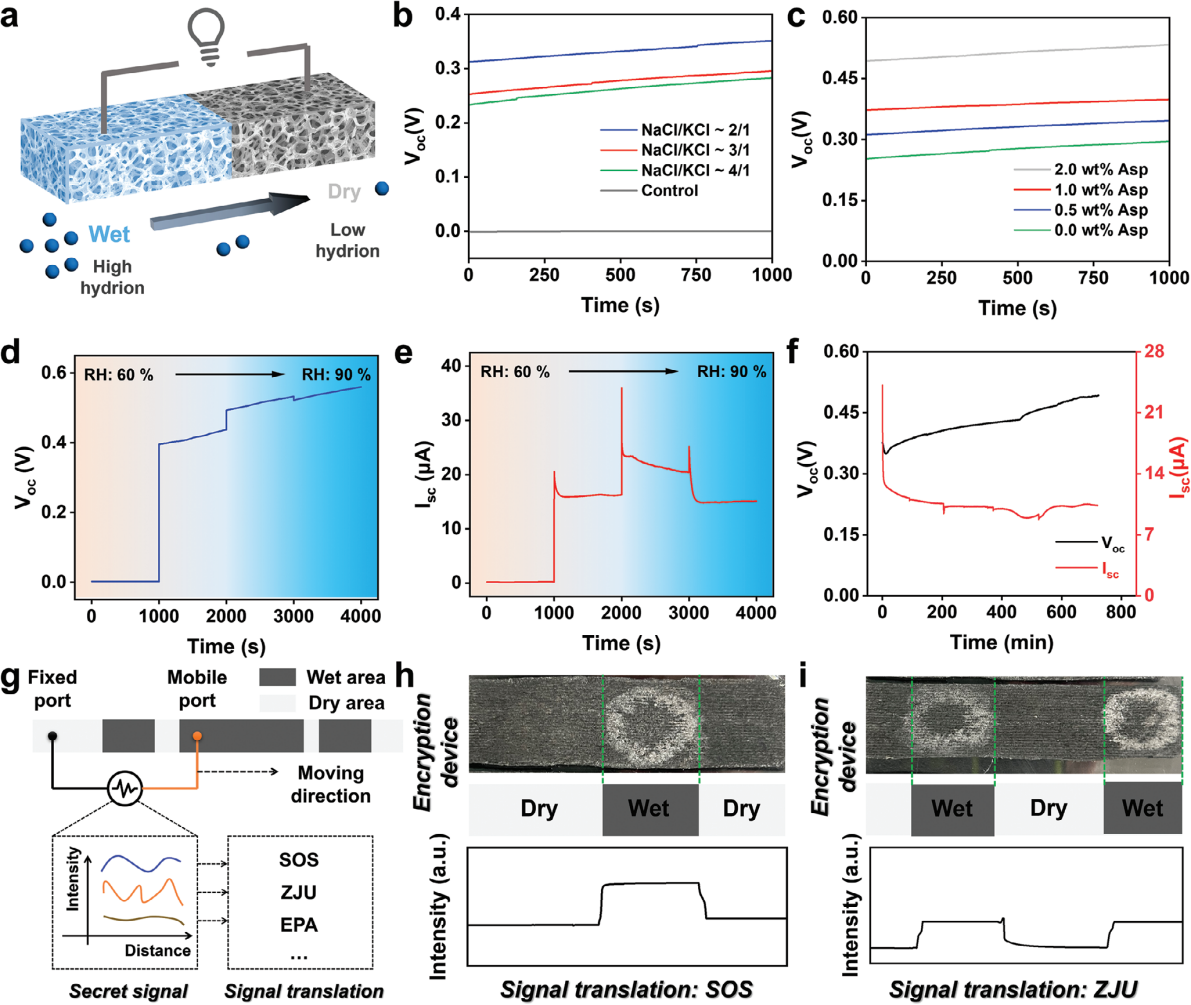
Moisture‐induced electricity generation performance of derived salt mixtures. a) Schematic diagram of the SMIEG; b) V_oc_ of the SMIEG prepared by different ratio of NaCl and KCl under 70% RH; c) V_oc_ of the SMIEG prepared by different ratio of mixed salts and L‐Asp under 70% RH; d) V_oc_ and e) I_sc_ of SMIEG under different RH. f) The long‐term V_oc_ and I_sc_ of SMIEG prepared by the extracted mixed salts. g) The schematic diagram of the SMIEG for the signal transmission, h,i) SMIEG carries different information.

The final step to treat fly ash washing leachate is the utilization of the extracted mixed salts in the above chapter. Here these mixed salts are used for moisture‐induced electricity generation. Likewise, the incorporation of L‐Asp further fortifies this process. The carbon black‐coated cloth was used as the substrate to fabricate the SMIEG, in which the extracted salts were coated on one side of the substrate (**Figure** **5**a). The carbon black‐coated cloth was fabricated by a simple repeated immersion method. Compared to pristine substrates (non‐woven fabrics), carbon black nanoparticles could be observed on the carbon black‐coated cloth (Figure S14, Supporting Information). Meanwhile, carbon black‐coated cloth presents super hydrophilicity (Inset in Figure S14, Supporting Information) and high surface potential (Figure S15, Supporting Information), ensuring that it serves as a good substrate to fabricate the SMIEG.

Given that the extracted salt is mainly composed of NaCl and KCl, we study the electrical performance of the SMIEG fabricated by the commercial salt under 70% RH (Figure 5b). The carbon‐black coated cloth performed a zero open circuit voltage (V_oc_), while SMIEG performed a significantly increased V_oc_. With the mass ratio of NaCl and KCl varying from 4/1 to 2/1, the V_oc_ escalated from 0.27 to 0.33 V. The increase on the V_oc_ may be related to the better moisture adsorption performance of KCl. The mass ratio of NaCl and KCl in the extracted salts is ≈2/1, so we utilized commercial NaCl and KCl with the same mixed ratio to assess the effect of L‐Asp. As expected, as the L‐Asp dosage increased from 0 wt% to 2 wt% of the mixed salts, the open circuit voltage (V_oc_) increased by 2 times (Figure 5c). This shows that L‐Asp can significantly enhance the moisture‐enabled electricity generation process.

Subsequently, we explored the electrical performance under different humidity conditions (Figure 5d,e). Consistent with the calculated results, the SMIEG showed no electrical performance under 60% RH. Note that the critical relative humidity of NaCl and KCl exceeds 75%, indicating the SMIEG derived from these compounds remains electrically dormant until this threshold is met. As mentioned above, despite L‐Asp's inability to enhance the salts' moisture absorption capacity, it effectively lowered the critical relative humidity (Figure 2c; Figure S16, Supporting Information). Therefore, with the RH increasing to 70%, the SMIEG showed an increased V_oc_ and short circuit current (I_sc_) of ≈0.42 V and 16.2 µA, respectively (Figure 5d,e). As the RH further increased to 90%, the V_oc_ also enlarged to 0.56 V (Figure 5d). In comparison, the I_sc_ of the SMIEG peaked at ≈21.6 µA under 80% RH (Figure 5e).

To further amplify the output performance, we adopted series and parallel connections here in 65% RH (Figure S17, Supporting Information). With the numbers of series and parallel connections increasing to 4, the V_oc_ and I_sc_ enlarged to 1.03 V and 20 µA, respectively (Figure S18, Supporting Information). To demonstrate the practical performance of the extracted mixed chlorides, we used the mixed salts extracted from washing leachate to prepare the SMIEG, the compositions were presented in Table S4 (Supporting Information). One can see that SMIEG enabled a stable V_oc_ and I_sc_ of ≈0.51 V and 10.6 µA within continuous 800 min, respectively (Figure 5f). Note that the V_oc_ gradually increases, while the I_sc_ remains essentially unchanged, which can be attributed to the charge accumulation and ion migration of SMIEG.^[^ ^28^ ^]^ Specifically, as SMIEG adsorbs moisture, positive charges (such as protons) accumulate on its surface, thus increasing the potential difference. Meanwhile, lacking strong external forces, such as intense light or wind speed, the migration rate of ions is constrained.^[^ ^29^ ^]^ In other words, the rate at which ions migrate from the interior to the surface may be slower than charge accumulation. As a result, the V_oc_ keeps rising in the above process. Additionally, the ion concentration gradient in the SMIEG may reach a dynamic equilibrium owing to the limited conductivity and ion mobility of SMIEG.^[^ ^30^ ^]^ Even if the V_oc_ rises, the I_sc_ cannot increase proportionally. In conclusion, the above results recommend the mixed salts with L‐Asp as a novel power source in real‐world applications.^[^ ^31^ ^]^

Indeed, most moisture‐induced electricity generators suffer from low power density (Eg, SM designed here is only 11 µW cm^−2^), which impedes their commercialization.^[^ ^32^ ^]^ In addition, the weak performance under low humidity also limits its application as a dynamo.^[^ ^33^ ^]^ In response, we have targeted SMIEG in signal transmission (Figure 5g). Due to the fast response speed and the high sensitivity to environmental humidity, SMIEG is a promising signal carrier. When one electrode moves from the dry area to the wet area while another electrode maintains stationary, SMIEG will experience different potential fluctuations. Therefore, SMIEG with the different wetted areas will reflect the different signals when controlling the moving frequency and speed. Like the Morse cipher, a password that carries specific information is constructed based on existing or self‐defined encryption logic, such as “SOS” and “ZJU” (Figure 5h,i).

### 2.5 Discussion

There were several issues needed to be further discussed: (1) Reusability of L‐Asp. In the whole technique route, we did not recycle L‐Asp. L‐Asp can be synthesized via the classic Strecker synthesis method or produced through fermentation.^[^ ^34^ ^]^ Given that L‐Asp is cost‐effective, widely available, environmentally friendly, and naturally biodegradable, we did not recycle L‐Asp. Additionally, we evaluated the cost benefits of using L‐Asp to produce vaterite CaCO_3_ (Details in Supplementary information). Even if L‐Asp cannot be recycled to use, using L‐Asp to induce the formation of vaterite CaCO_3_, can also obtain higher profits ($212.42 per ton of vaterite). (2) Substitutability of L‐Asp. In our carefully designed processing route (Figure S19, Supporting Information), the primary roles of L‐Asp are to promote the formation of vaterite, enable stable salt crystallization, and enhance the electricity generation performance of the resulting salts. While other hygroscopic amino acids, such as glycine and glutamic acid, may also contribute to moisture‐enabled electricity generation, they cannot achieve the other two functions. Thus, we did not compare the enhanced effects of different amino acids here. Further research can be conducted in the future. Meanwhile, our results show that L‐Asp does indeed improve electricity generation performance, with the open circuit voltage (V_oc_) doubling as the L‐Asp content increased from 0 to 2 wt% in the mixed salts. (3) Flexible applicability of L‐Asp. The technical route scheme for treating the fly ash is shown in Figure S19 (Supporting Information). To prepare more valuable vaterite CaCO_3_, we have added excess L‐Asp in the early stage. Since L‐Asp is not removed during the whole process, it will remain in the resulting high‐salinity wastewater and as‐obtained salt crystals, thus enhancing salt extraction and SMIEG easily. Adding excess L‐Asp at the early stage of the high‐salinity wastewater treatment route allows it to play a role throughout the entire process, giving L‐Asp flexible applicability.

## 3 Conclusion

In summary, we have verified the positive effect of the L‐Asp for the treatment of the high‐salinity brine, taking fly ash washing leachate as an example. DFT calculation‐guided experiments implied that L‐Asp could promote the transformation from cheap calcite to valuable vaterite during the softening process (pretreatment before crystallization). Then solar‐driven interfacial evaporator was employed to treat the as‐prepared solution, with a stable evaporation rate of 1.20 kg m^−2^ h^−1^ and a salt recovery ratio of 0.14 kg m^−2^ h^−1^ within 40 h under 1 sun. Like distorting the crystal structure of calcium carbonate, L‐Asp also has a similar ability to inorganic salts, thereby making the mixed chloride salt more porous and easier to separate. Finally, to harness the “cradle to grave” full life cycle utilization of washing leachate, the extracted mixed salts are employed for moisture‐induced electricity generation. L‐Asp can significantly enhance this process by reducing the critical relative humidity of mixed salts. Given the low electricity output of a moisture‐induced electricity generator, we showed its huge potential as a signal carrier. In conclusion, L‐Asp with dual functions could promote the comprehensive utilization of high‐salinity wastewater, also promoting the Sustainable Development Goals.

## 4 Experimental Section

### Materials and Regents

The MSWI fly ash sample was obtained from the waste incineration plant (Ningbo, China). MSWI fly ash is mainly composed of CaSO_4_, CaCl_2_, Ca(OH)_2_, CaCO_3_, NaCl, and KCl. The detailed composition of MSWI fly ash was shown in Table S1 (Supporting Information). The sintering dust washing leachate was obtained from the sintering dust disposal production line of Taigang company (Taiyuan, China). The main composition was shown in Table S2 (Supporting Information). The carbon black was bought from Acme (China). Other reagents such as Na_2_CO_3_, L‐Asp, and HNO_3_ were bought from Macklin (China). The chemicals were all analytical grade and used without further purification. The activated carbon cloth, polyethylene Styrene foam (PS foam), non‐woven fabrics, and alligator clip wires were from Alibaba (China).

### Acid Pickling of MSWI Fly Ash

Before the washing experiment, the original MSWI‐FA was homogenized in a plastic bucket and vacuum dried at 65 °C for 12 h. In a typical batch, 5 g of the original MSWI‐FA sample was mixed with 15 ml of HCl (0–4 m) in a covered glass beaker. The mixture was stirred at 900 rpm for 120 mins. Subsequently, the mixture was filtered using a vacuum filtration system with a 0.45‐micron filter membrane to separate the liquid and solid phases. The concentrations of metals in the pickling leachate were analyzed through Inductively Coupled Plasma‐Atomic Emission Spectrometry (ICP‐AES).

### L‐Asp Controlled Vaterite Production

A certain amount of L‐Asp (0–10 mM) was added to the MSWI fly ash washing leachate before wet carbonation. The liquid‐to‐solid ratio (L/S) of the above mixtures was 3. 1 m Na_2_CO_3_ solution was dropwise added to the leachate under continuous magnetic stirring until the pH value increased over 11. Subsequently, the CaCO_3_ was obtained through vacuum filtration. The precipitate was washed with deionized water for several times and dried at 65 °C overnight. The derived CaCO_3_ was labeled as APA (Acid pickling with L‐Asp addition). For comparison, the MSWI fly ash washing leachate was carbonated with Na_2_CO_3_ without L‐Asp regulation and the derived CaCO_3_ was labeled as AP. The CaCO_3_ derived from the system without pH control and L‐Asp addition was labeled as CG.

### Solar‐Driven Salts Extraction

The classical single‐flowing solar evaporator (CSFS, Figure 4a) was used to treat the decalcified leachate, while a traditional solar evaporator (Figure S11, Supporting Information) with uniform surface water supply was selected for comparison. The fabricated details of these two evaporators were provided in Supplementary Information. The evaporation performance was measured on a homemade optical system.^[^ ^35^ ^]^ The Xenon lamp (CHF‐XM500, China), equipped with an AM 1.5 G filter, was utilized to simulate sunlight. The solar flux was calibrated using a light intensity meter (Spectronics‐3000 UV‐AB). The mass change of CSFS under solar irradiation was used to calculate the evaporation rate.^[^ ^36^ ^]^ The salt‐extraction performance was studied under 10.0 wt% leachate with variable L‐Asp dosages. The as‐obtained salt was dried in an oven at 105 °C for further analysis.

### Moisture‐Induced Electricity Generation

0.2 g of carbon black and 0.4 g of dodecyl ammonium bromide were dispersed into 40 ml of deionized water under an ultrasonic container, thereby obtaining carbon black suspension. The non‐woven fabric with an area of 1 cm × 6 cm was coated with carbon black suspension and then dried at 60 °C. After repeating several times, the carbon black‐coated cloth was obtained until the non‐woven fabric strips were uniformly loaded with a sufficient amount of carbon black. NaCl, KCl, and L‐Asp were used to prepare salt solutions with different components. A certain amount of the prepared solution was dropped on the one side of the prepared carbon black‐coated cloth, and then dried at 85 °C. This procedure was repeated several times until the mass loading of the salts reached 0.05 g cm^2^. In this way, a simple and stable salts‐based moisture induced electricity generator (SMIEG) was obtained. The SMIEG with different salt loads was fabricated according to the experimental design. The open circuit potential (V_oc_) and short‐cut current (I_sc_) between the dry and wet sides of the SMIEG were measured through an electrochemical workstation (CHI660e).^[^ ^37^ ^]^ The humidity during the test is controlled by a constant temperature and humidity chamber (HWS‐50).

## Conflict of Interest

The authors declare no conflict of interest.

## Author Contributions

S.M. and Z.Y. contributed equally to this work. C.S. and X.L. conceived and designed the project; S.M. and Z.Y. did the experiments; Z.Y. contributed to the theoretical analysis; S.M. and Z.Y. organized the data and wrote the manuscript. All authors discussed the results and approved the final version of the manuscript.

## Supporting information



Supporting Information

## Data Availability

The data that support the findings of this study are available from the corresponding author upon reasonable request.
