# Supplementary material for: L‐Aspartic Acid with Dual Functions: An Eco‐Friendly and Affordable Choice to Accelerate High Salinity Brine Utilization
Source: Adv Sci (Weinh). 2025 Jan 21;12(10):2408081. doi: 10.1002/advs.202408081 (PMC11904999; doi:10.1002/advs.202408081)
Supplement: Supplementary file 1 — Supporting Information [file ADVS-12-2408081-s001.docx]

Supplementary Information for

**L-Aspartic Acid with Dual Functions: an Eco-Friendly and Affordable Choice to Accelerate High Salinity Brine Utilization**

Suning Mao^a#^, Zhen Yu^a#^, Jie Chen^a^, Yizhe Shen^a^, Minjie Li^a^, Chen Sun^b,c*^, Xiaoqing Lin^a*^, Bin Yang^c^, Tong Chen^a^, Qunxing Huang^a^, Xiaodong Li^a^, Jianhua Yan^a^

^a^ State Key Laboratory of Clean Energy Utilization, Institute for Thermal Power Engineering, Zhejiang University, Hangzhou 310058, China

^b^ Institute of Zhejiang University - Quzhou, No. 99 Zheda Road, Quzhou 324000, China

^c^ Key Laboratory of Biomass Chemical Engineering of Ministry of Education, College of Chemical and Biological Engineering, Zhejiang University, Hangzhou 310027, China

^*^Corresponding author: sunchen1@zju.edu.cn (Chen Sun) and linxiaoqing@zju.edu.cn (Xiaoqing Lin).

^#^Suning Mao and Zhen Yu contributed equally to this work.

**1** **Supplementary Method**

**1.1 Fabrication of two evaporators**

The two evaporators both consist of an absorber, water transfer channel, insulation material, and water storage container. Super hydrophilic carbon felt with a diameter of 5 cm and polystyrene foam (PS foam) with a diameter of 4 cm were used as absorbers and insulation layers, respectively. The CSFS is made as follows: a circular super-hydrophilic carbon felt with a diameter of 5 cm is placed on top of the PS foam, and a long strip of carbon felt with a diameter of 5 mm is used as a central water transport channel through the PS foam. By sequentially combining and fixing the absorber, PS foam, and long strips of carbon felt into a container, a classical single-flowing structure was obtained. The traditional evaporation structure is made the same except for the water supply channel replacing by a non-woven fabric which wraps with PS foam. Both ends of the non-woven fabric are immersed in the evaporation mother liquor.

**1.2 Material characterization**

The morphology was characterized by a scanning electron microscope (SEM, ZEISS Gemini SEM 300, Germany). The contact angles were measured by the dynamic contact angle tester (OCA20, Germany). The optical absorption was analyzed by a UV-*vis*-NIR spectrophotometer (UV-3101, Japan) equipped with an integrating sphere. The ZETA potential was recorded by a particle sizer analyzer (Zeta sizer Nano ZS90, Malvern Instruments, UK). Inductively coupled plasma optical emission spectroscopy (ICP-OES, VARIAN 730-ES, Austria) was used to measure the concentrations of metals in the washing leachate. The XRF analysis of the raw ingredients and derived salts was performed on an X-ray fluorescence spectrometer (S8 Tiger, Bruker, Germany). The CaCO_3_ samples are identified by the X-ray diffraction (XRD) method (Bruker D8 ADVANCE) from 10°-80° at a rate of 3° min^-1^.

**1.3 DFT calculation**

Density functional theory (DFT) calculations were carried out using the Vienna Ab-initio Simulation Package (VASP).[1] The whole system contains a complete graphical interface for setting up, running, and analyzing VASP calculations. The projected augmented wave (PAW) potential was employed to describe the core electrons, and the calculations were performed within the framework of the generalized gradient approximation (GGA), specifically using the Perdew-Burke-Ernzerh (PBE) functional to calculate the exchange-correlation energy.[2] The plane wave basis set was extended with a cut-off energy of 500 eV. A 3 × 3 × 1 Monkhorst Pack *k*-point grid was utilized for structure optimizations and electronic structure calculations. The conjugate gradient method was employed to fully optimize the positions of the system until all forces on each atom were reduced to less than 0.02 eV/Å. The energy convergence of the whole self-consistent process was determined based on 10^-5^ eV. The Gaussian smearing broadening was set to 0.05 eV.[3]

- 1. **Cost analysis of salt extraction process**

Before calculation, we made the following assumptions:

1. The amount of high-salinity wastewater is set as 100 mL, and the salinity is about 10%. The addition of L-Asp is 2 mM.
2. All the salt in the high-salinity wastewater is recovered (~10 g salts).
3. The working condition is 1 kW m^-2^, and the crystallization time is 8 hours.
4. The electricity cost or labor cost required to fabricate large-scale devices is negligible.

The price of raw materials and reagents was listed as follows：

| **Materials or Reagents** | **Unit Price** |
| --- | --- |
| Carbon felt | $ 21.2 m^-2^ |
| PS foam | $ 1.6 m^-2^ |
| L-Asp | $ 0.137 kg^-2^ |

To crystallize all the high-salinity wastewater within the set time in assumption, a 0.01 m^-2^ of evaporator is required. The detailed cost is calculated as follows:

| **Materials or Reagents** | **Unit Price** | **Amount** | **Total Price** |
| --- | --- | --- | --- |
| Carbon felt | $ 21.2 m^-2^ | 0.012 m^-2^ | $ 0.254 |
| PS foam | $ 1.6 m^-2^ | 0.01 m^-2^ | $ 0.016 |
| L-Asp | $ 0.137 kg^-2^ | 0.2662 g | $ 0.00003 |
| Total | - | - | $ 0.270 |

Therefore, the cost of salt extraction process is $ 0.0270 g^-1^ salts.

**1.5 Economic Analysis of CaCO_3_** **value-added**

Before calculation, we first explained the research background:

1. The price of vaterite CaCO_3_ (size: 0.1 μm) is $314 per ton, while the price of calcite CaCO_3_ of the same size is only $55 per ton;
2. The price of L-Asp is $137 per ton;
3. To produce one ton of vaterite CaCO_3_, 340 kg of L-Asp is needed;

The cost gain of producing vaterite CaCO_3_ is calculated as follows:

| CG=C_1_-C_2_-C_3_ | **(Equation S1)** |
| --- | --- |

Where C_1_ is the price of vaterite CaCO_3_, C_2_ is the price of calcite CaCO_3_, and C_3_ is the cost of the added L-Asp ($46.58). Based on **Equation S1**, the cost gain is calculated to be $212.42. Given that the addition amount of L-Asp is little, even if L-Asp cannot be recycled to use, using L-Asp to induce the formation of vaterite, can also obtain higher profits ($ 212.42 per ton of vaterite).

**1.6 Statistical Analysis**

Statistical analysis was done in Origin 2024b (Learning Edition), and statistical signiﬁcance was attributed to values of P < 0.05 as determined by Student’s t-test or one-way ANOVA analysis, as described in the ﬁgure legends. All data, are representative of three or more independent experiments and expressed as mean ± SEM.

**2 Supplementary Figures and Tables**

**Figure S1.** pH variation under different HCl dosages during acid pickling of MSWI fly ash


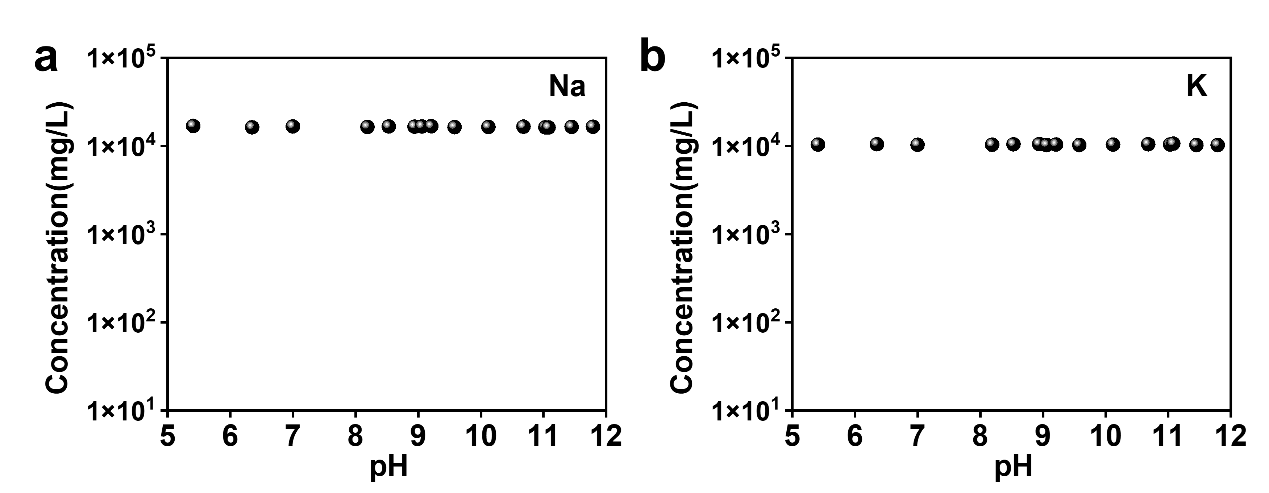


**Figure S2.** Na and K concentrations in the acid pickling leachate under different pH values


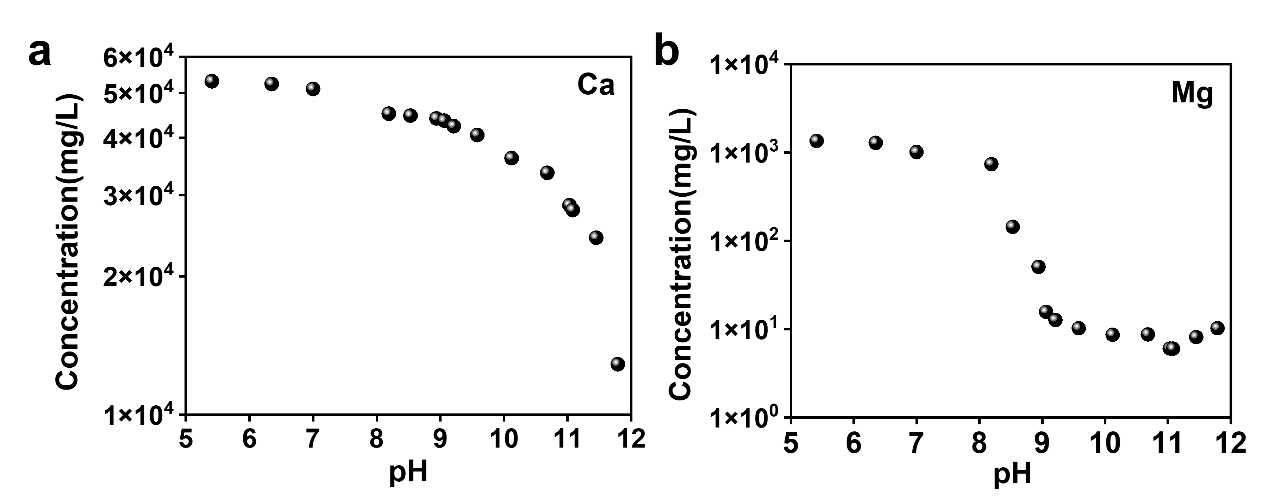


**Figure S3.** (a) Ca and (b) Mg concentrations in the acid pickling leachate under different pH values.


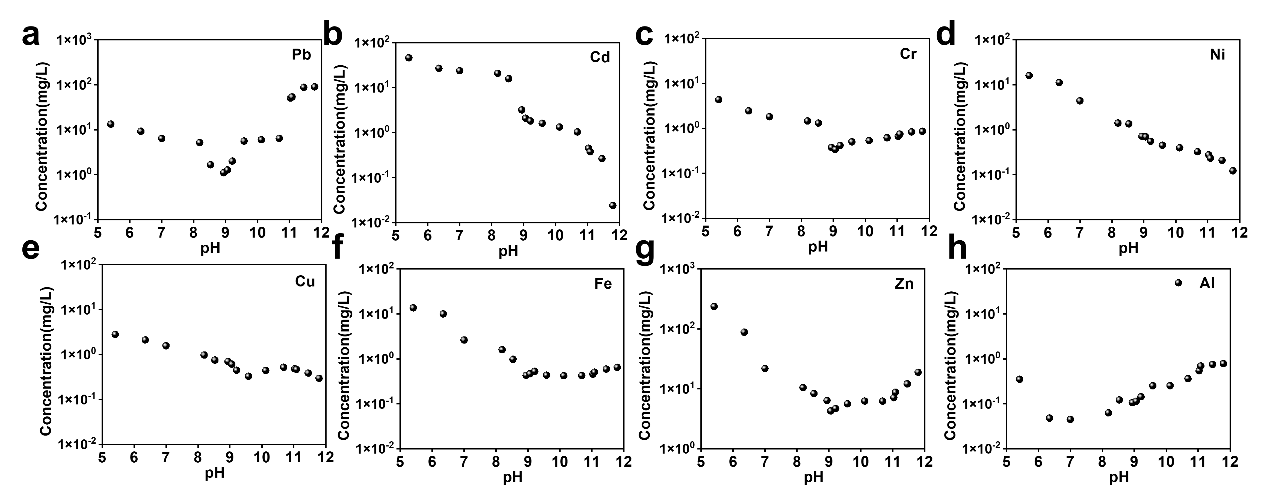


**Figure S4.** Heavy metals concentrations in the acid pickling leachate under different pH values. (a) Pb, (b) Cd, (c) Cr, (d) Ni, (e) Cu, (f) Fe, (g) Zn, and (h) Al.


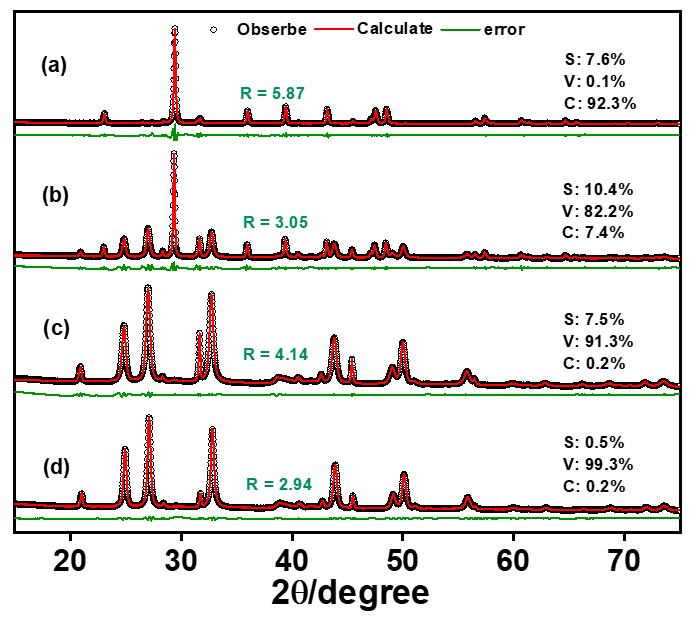


**Figure S5.** Fitting results of XRD patterns of CaCO_3_ precipitates derived from L-Asp regulated wet carbonation. (a) 0 mM; (b) 2 mM; (c) 5 mM; and (d) 10 mM.


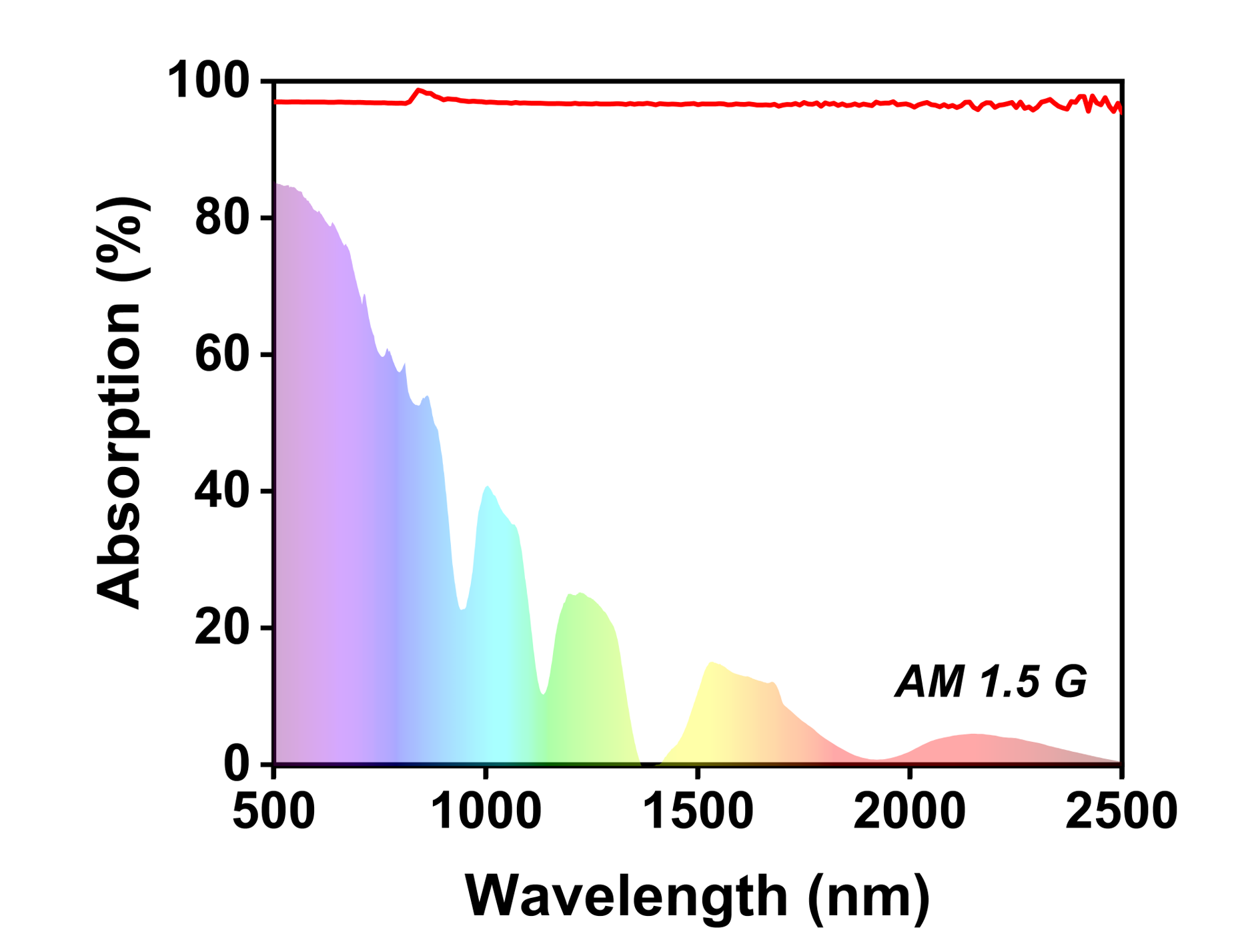


**Figure S6.** The UV–Vis-NIR absorption spectra of carbon felt.


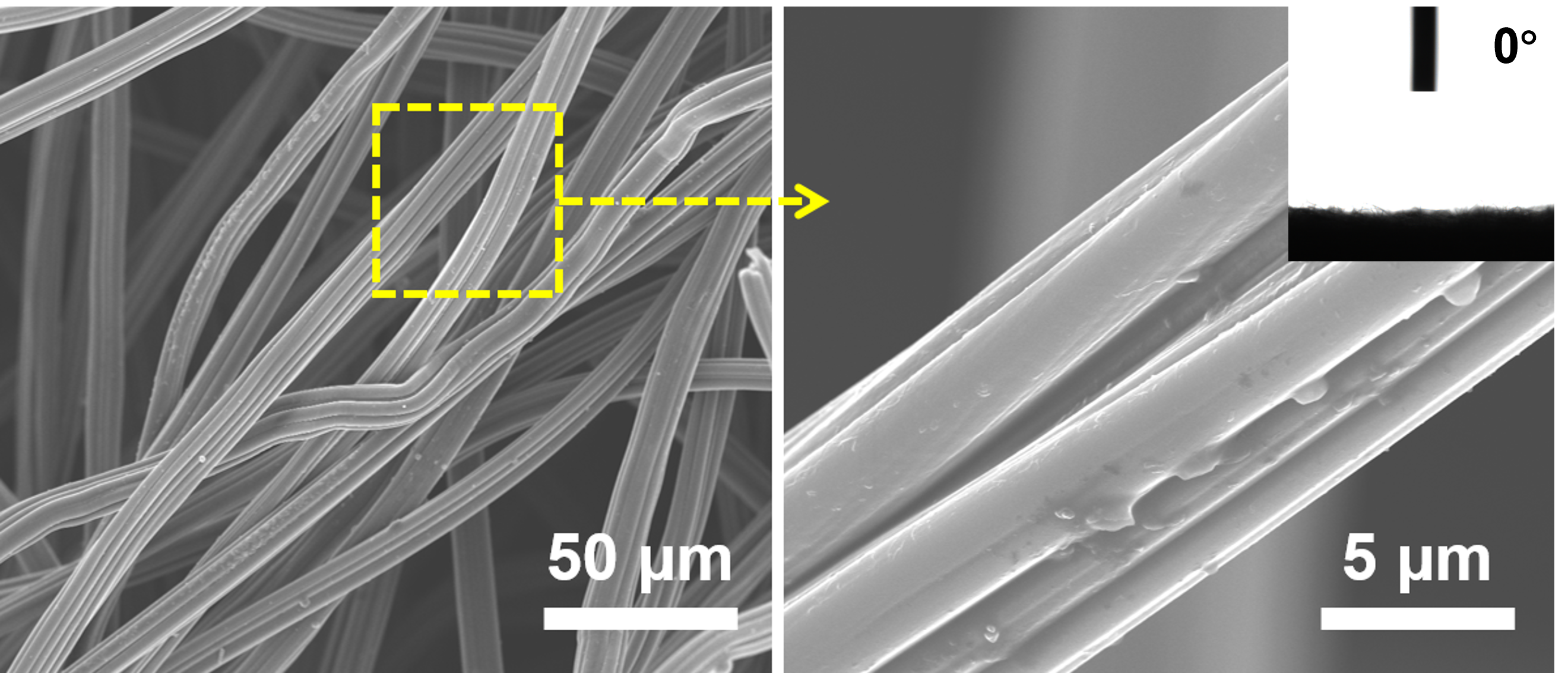


**Figure S7.** SEM images of carbon felt. Inset: The contact angle between water and carbon felt, showing the super-hydrophilic ability of carbon felt.


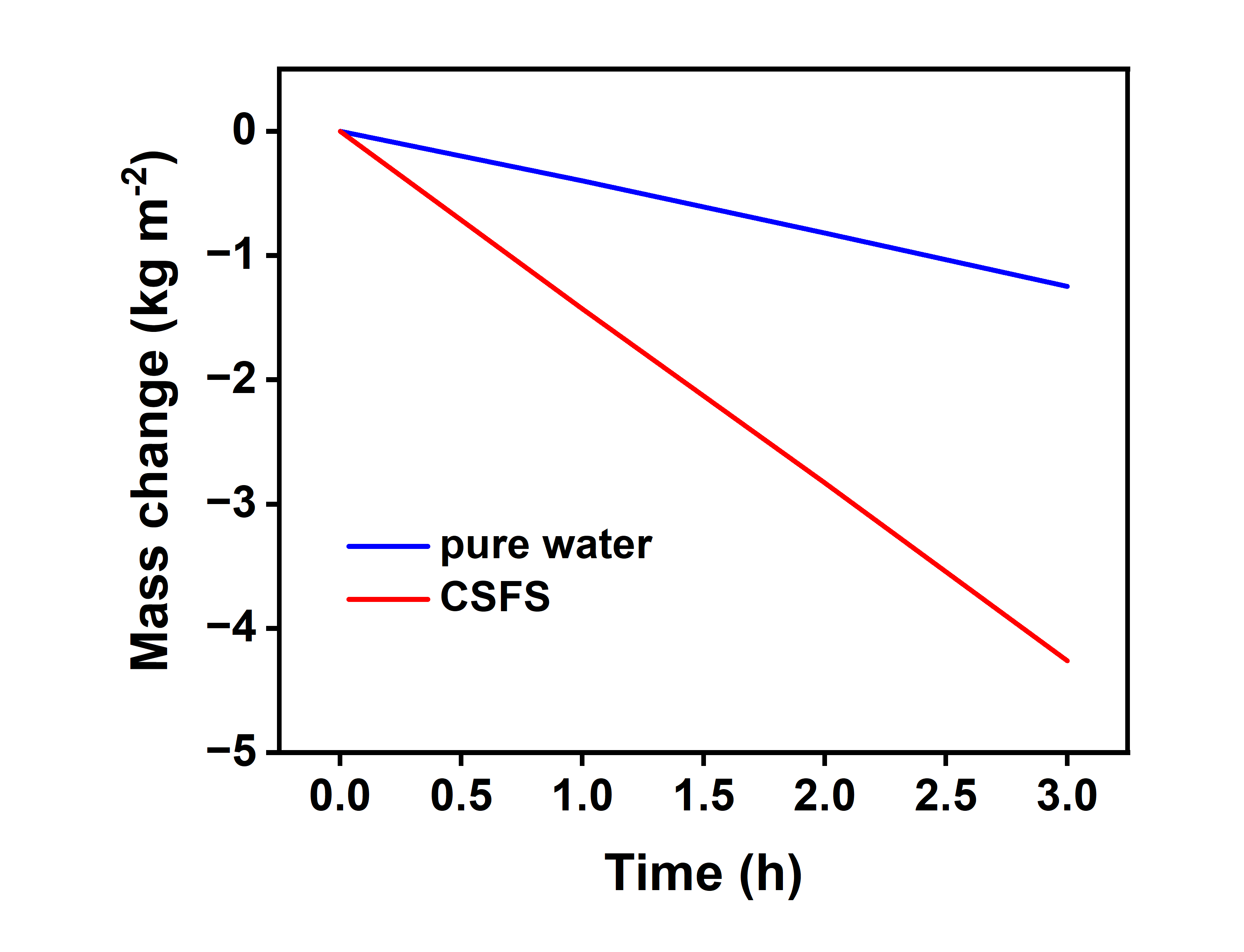


**Figure S8.** Mass change of pure water and CSFS under 1 sun.


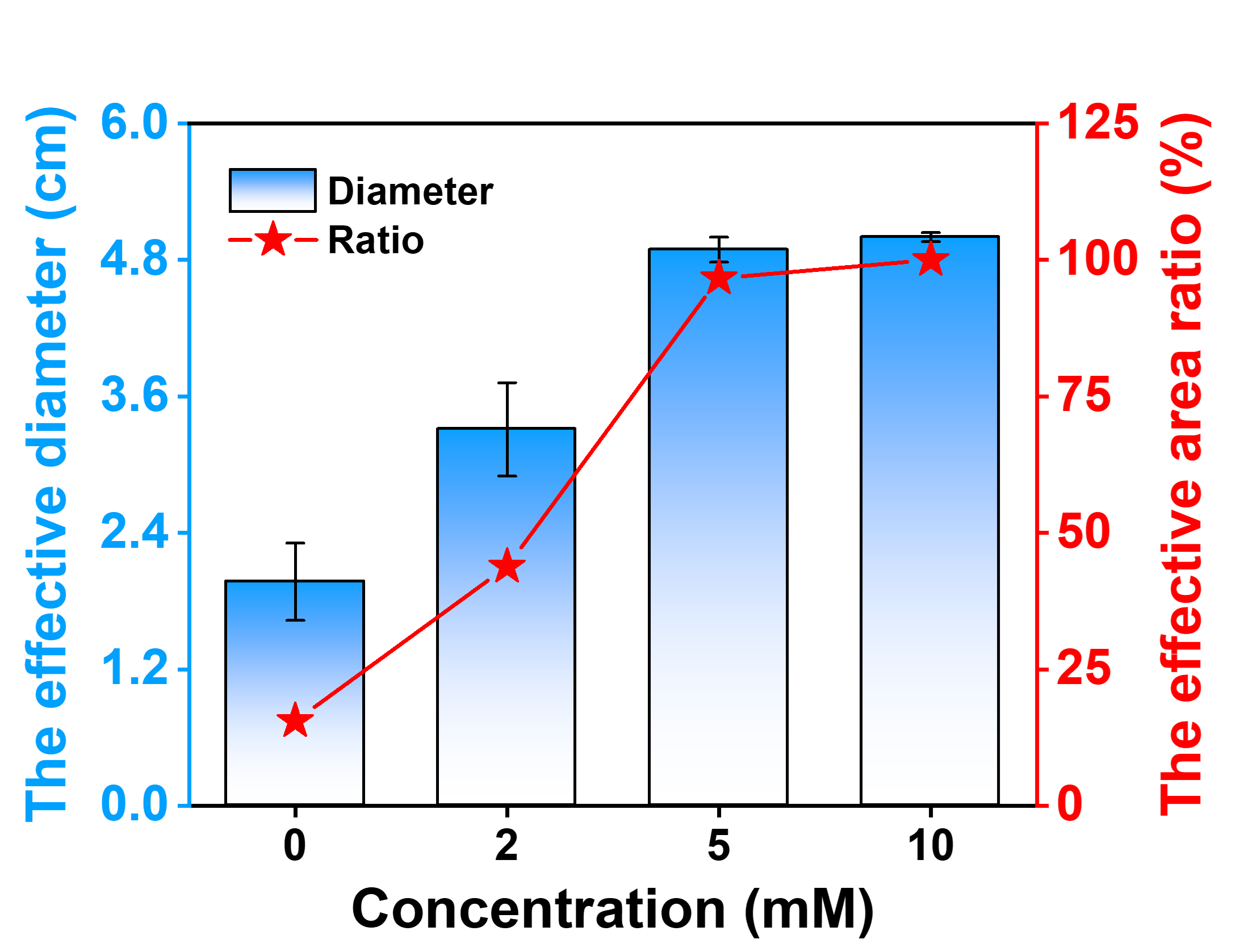


**Figure S9.** Effective evaporation diameter and effective area ratio of CSFS in the fly ash washing leachate with different L-Asp dosages.


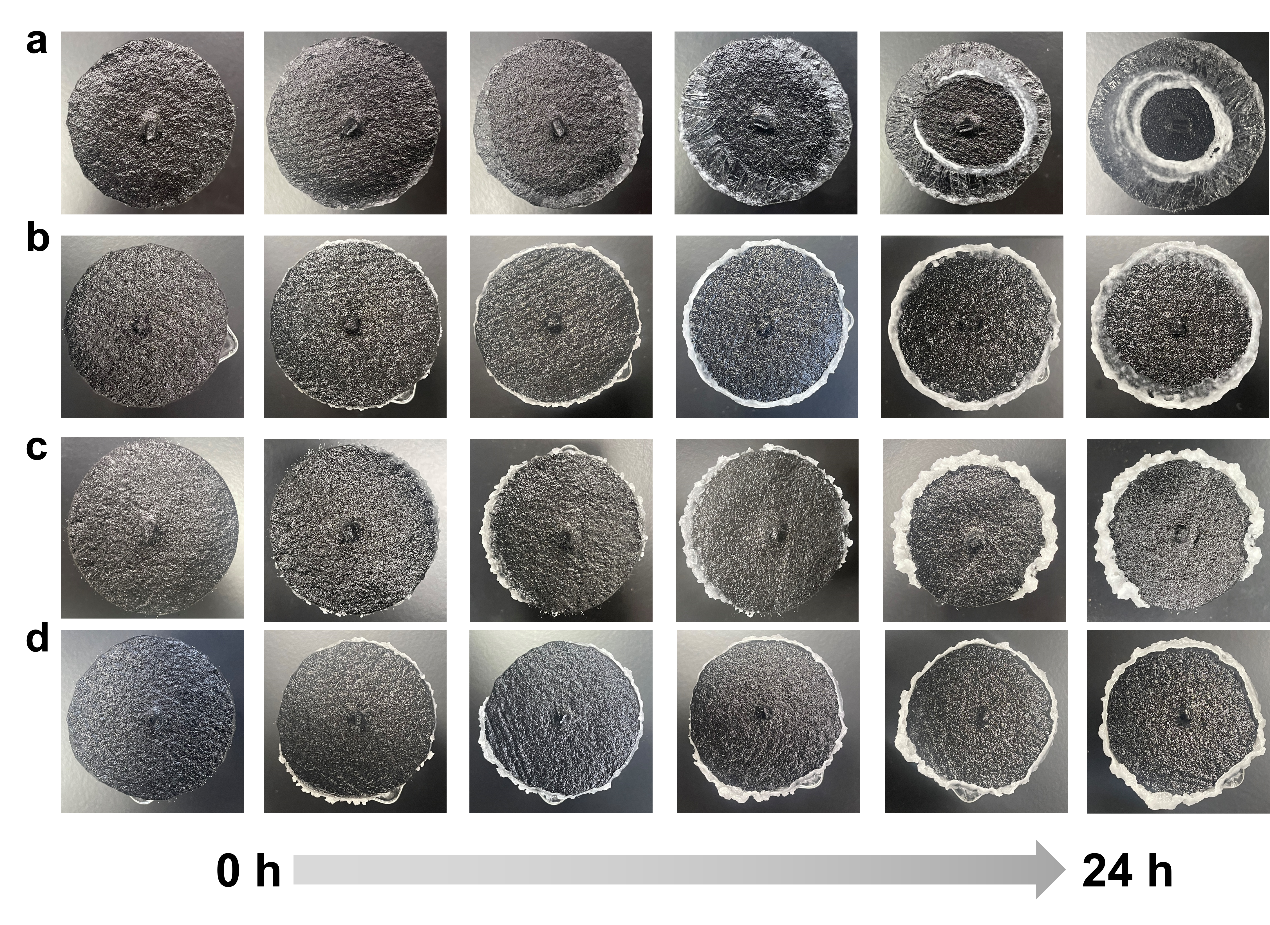


**Figure S10**. Top view photos of CSFS during solar-driven salts extraction process from MSWI fly ash leachate under different L-Asp dosages. (a) 0 mM; (b) 2 mM; (c) 5 mM; and (d) 10 mM.


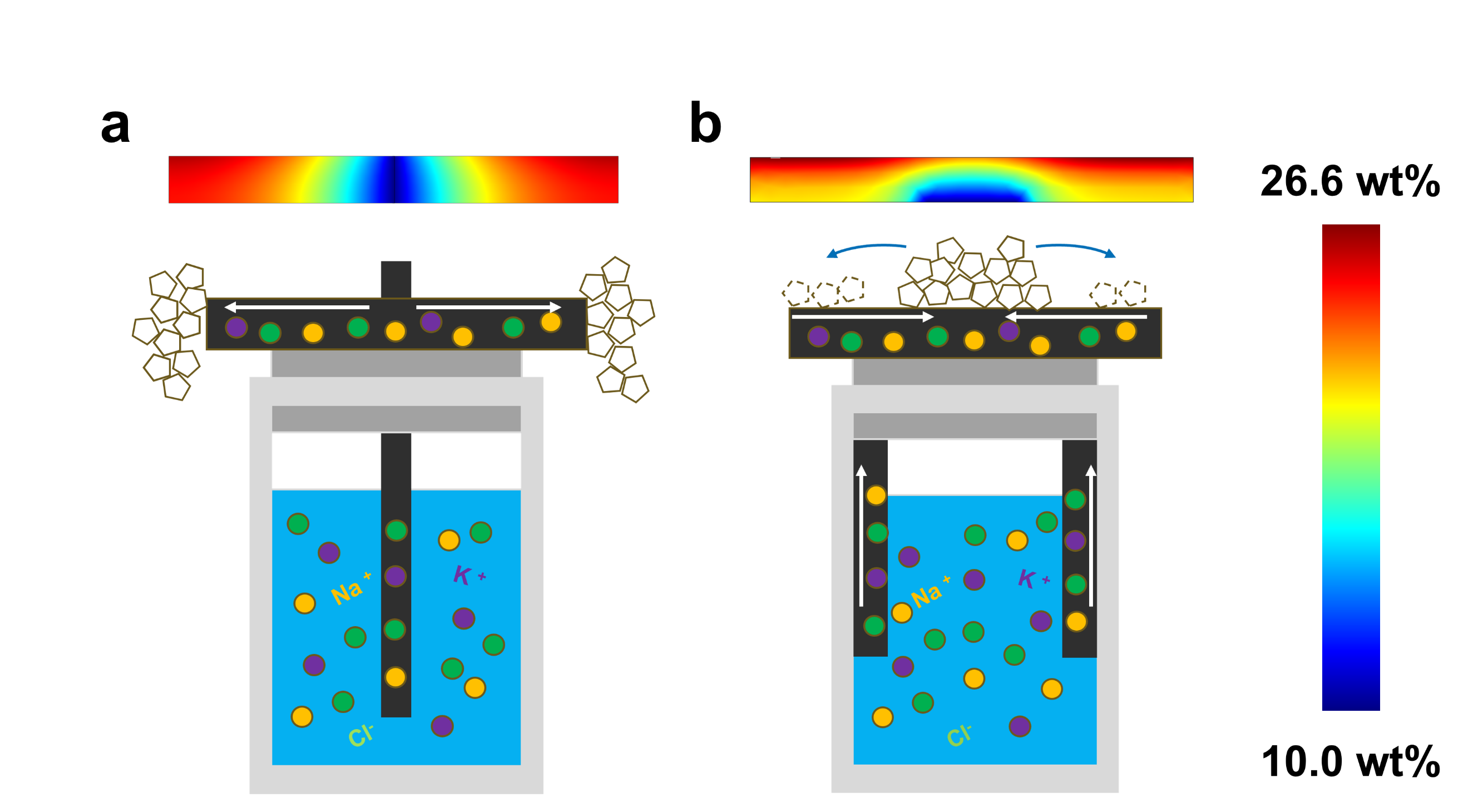


**Figure S11.** Absorber’s salts distribution simulated by COMSOL software and schematic diagram of (a) CSFS and (b) traditional solar evaporator.


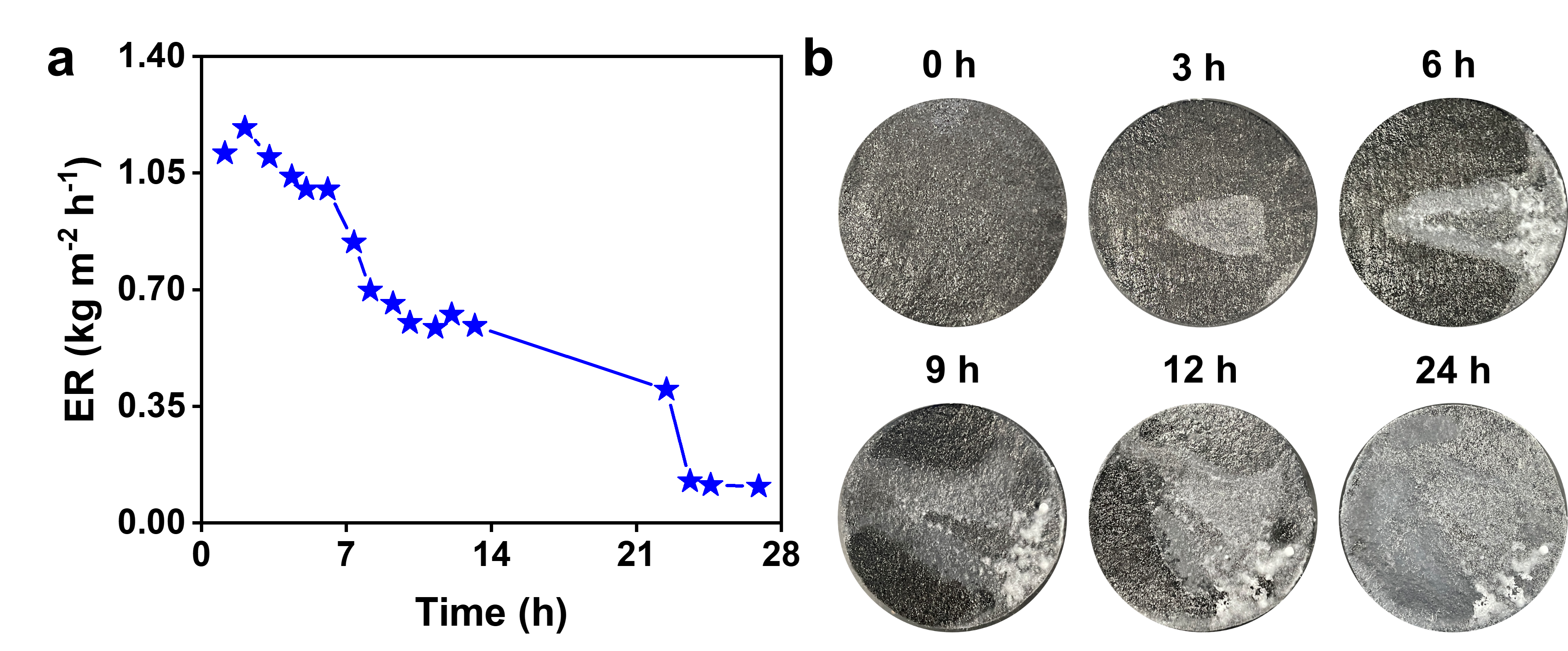


**Figure S12.** The desalination performance of traditional solar evaporator under 1 sun. (a) The evaporation rate (ER); (b) Top view photos during solar-driven salts extraction process.


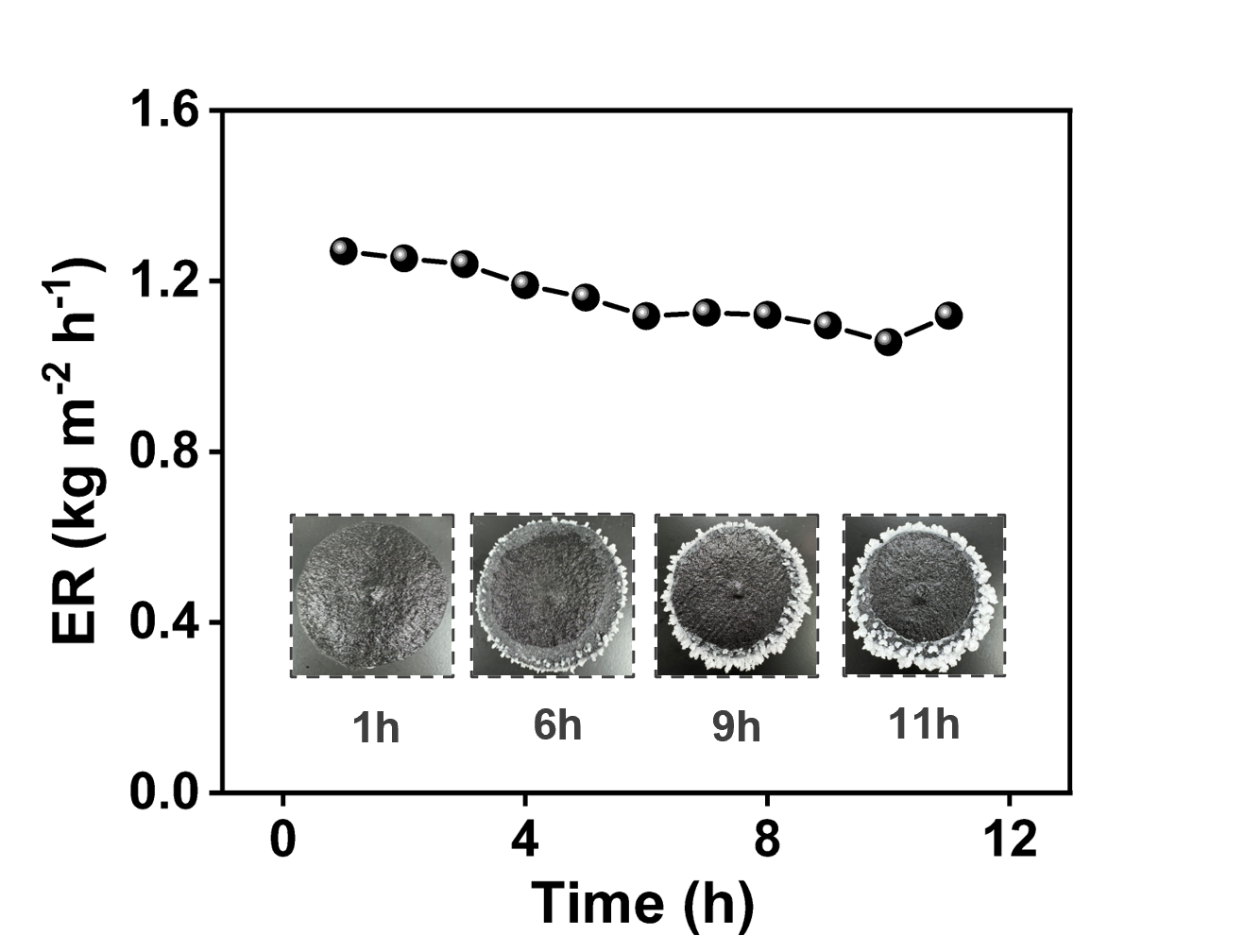


**Figure S13.** Stable solar-driven salts harvesting assisted by L-Asp on sintering dust washing leachate (L-Asp: 10 mM)


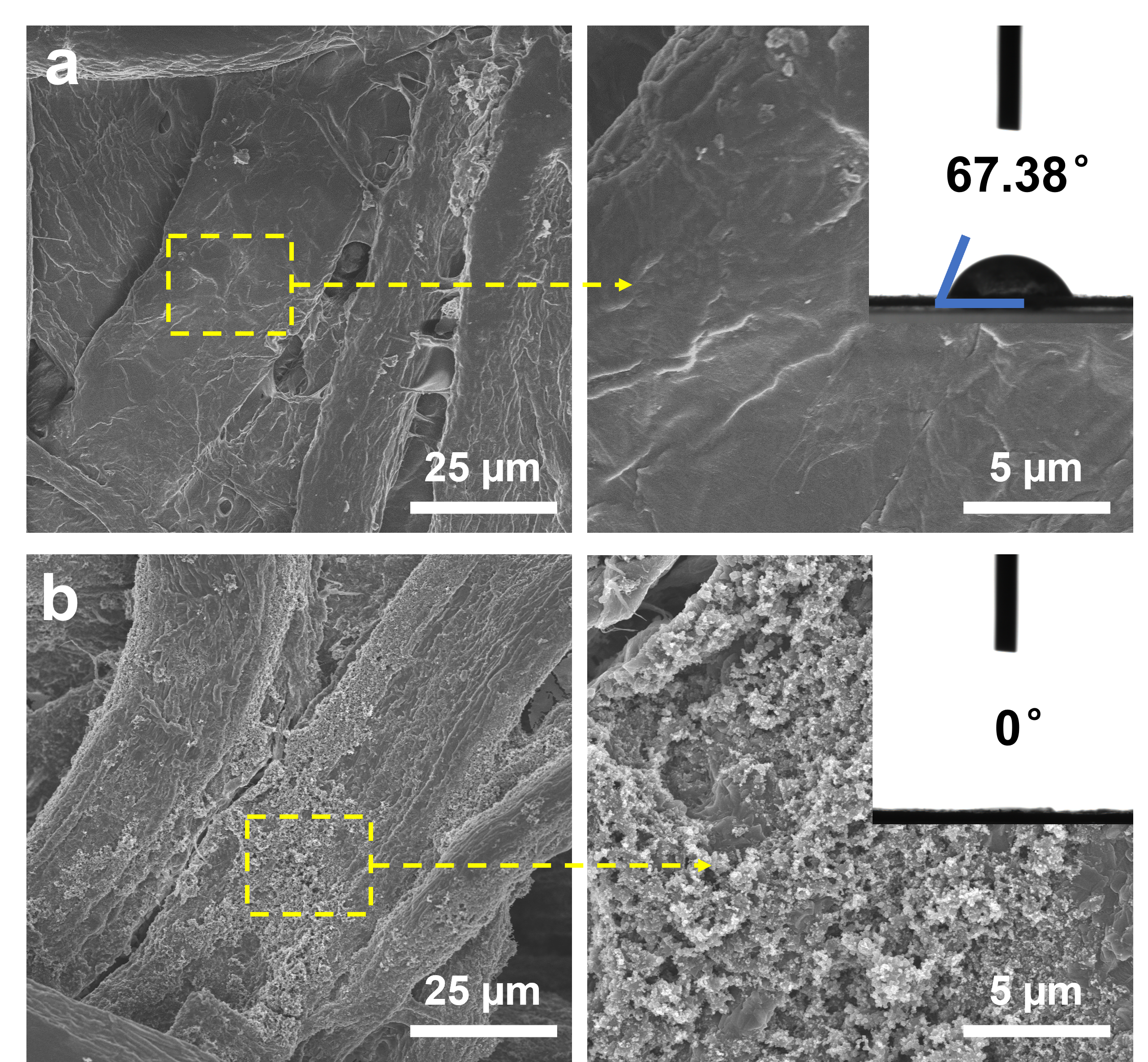


**Figure S14.** SEM images: (a) non-woven fabrics and (b) carbon black loaded cloth. Inset: The contact angle between water and (a) non-woven fabrics and (b) carbon black loaded cloth.


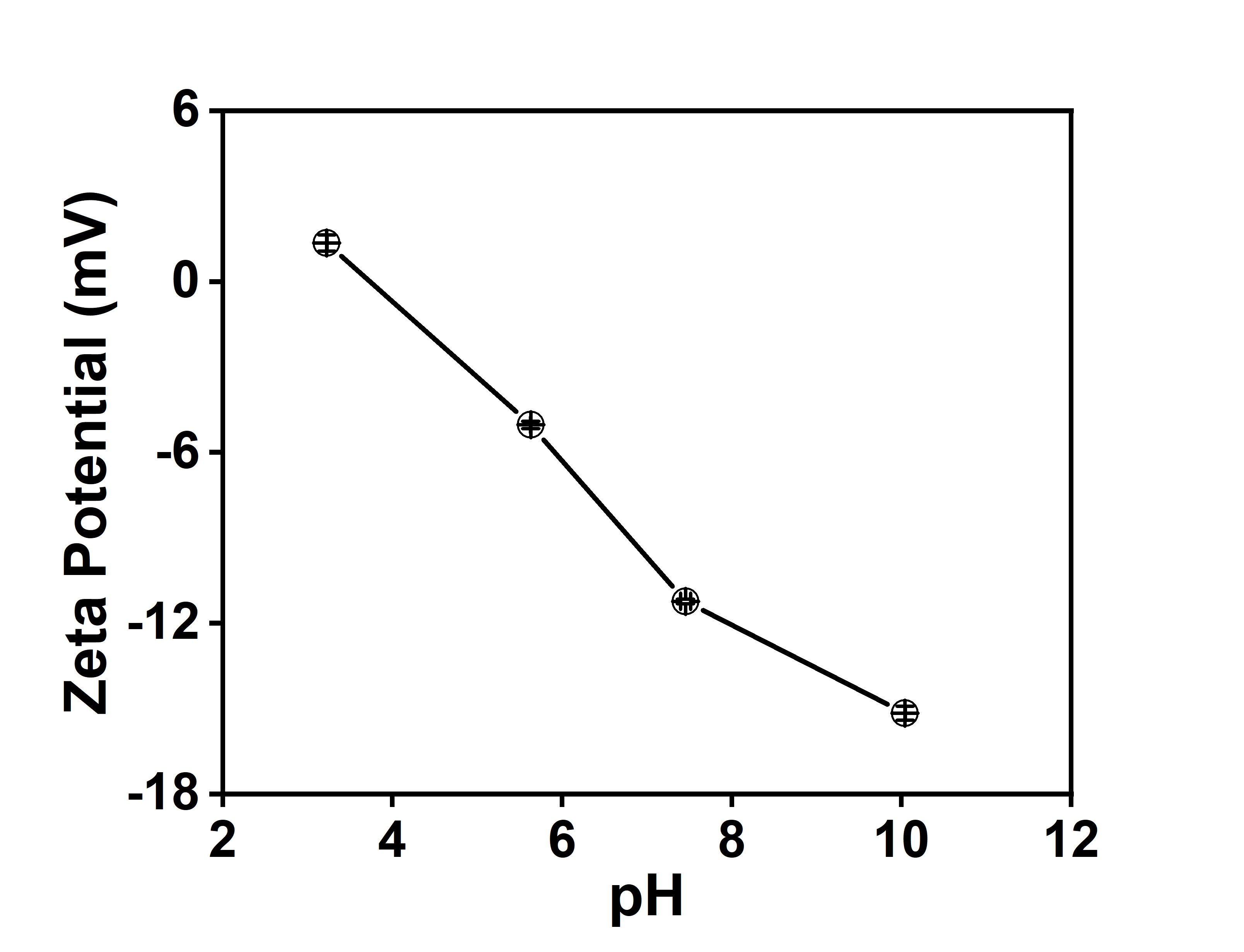


**Figure S15.** Zeta potential of carbon black loaded cloth under different pH values


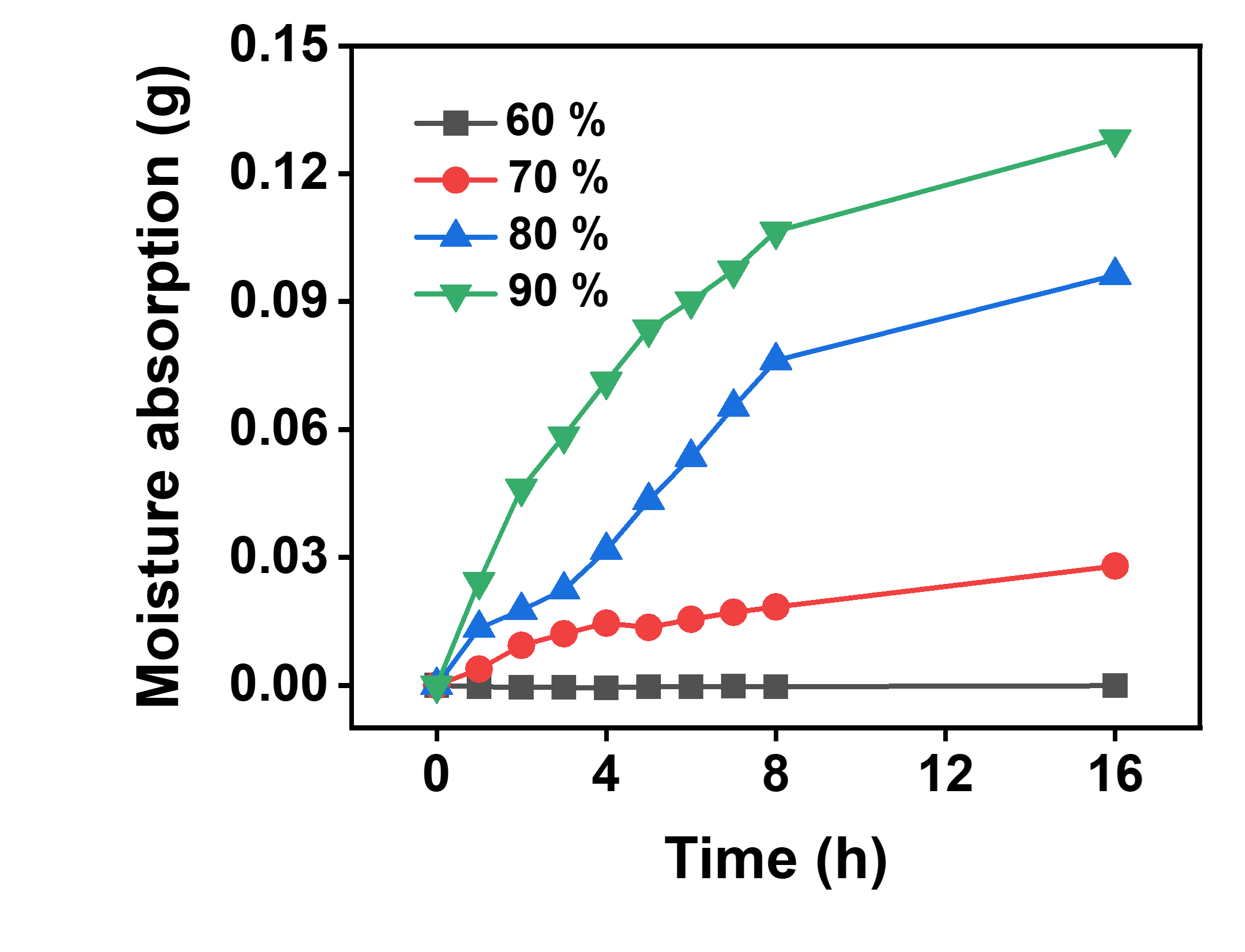


**Figure S16.** Moisture-adsorption amounts of L-Asp modified salt mixture under different relative humidities. The above-mentioned mixed salt can absorb moisture under RH 70%, showing L-Asp's ability to lower the critical relative humidity effectively.


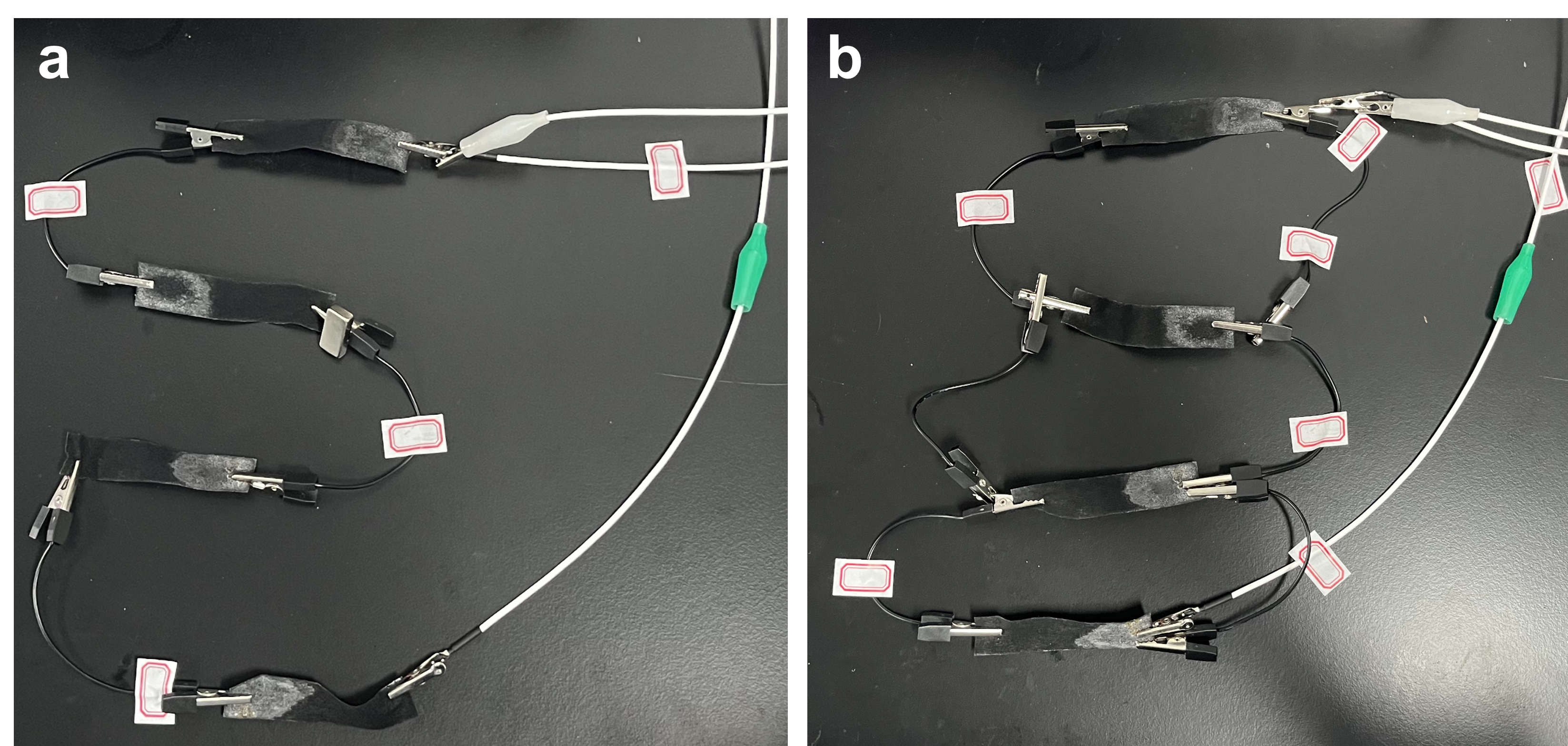


**Figure S17.** Photos of (a) series-connected and (b) parallel-connected SMIEGs


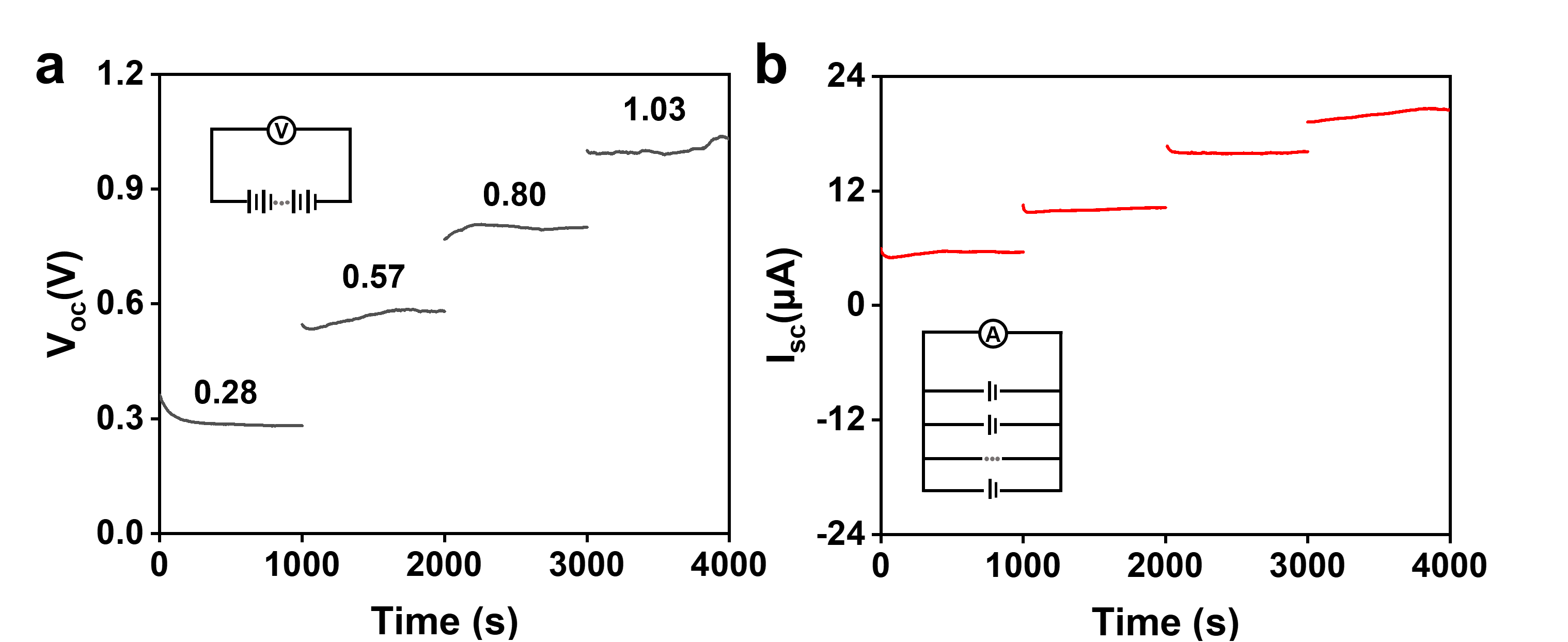


**Figure S18.** (a) V_oc_ of 1, 2, 3, and 4 series-connected SMIEGs; (b) I_sc_ of 1, 2, 3, and 4, parallels-connected SMIEGs.


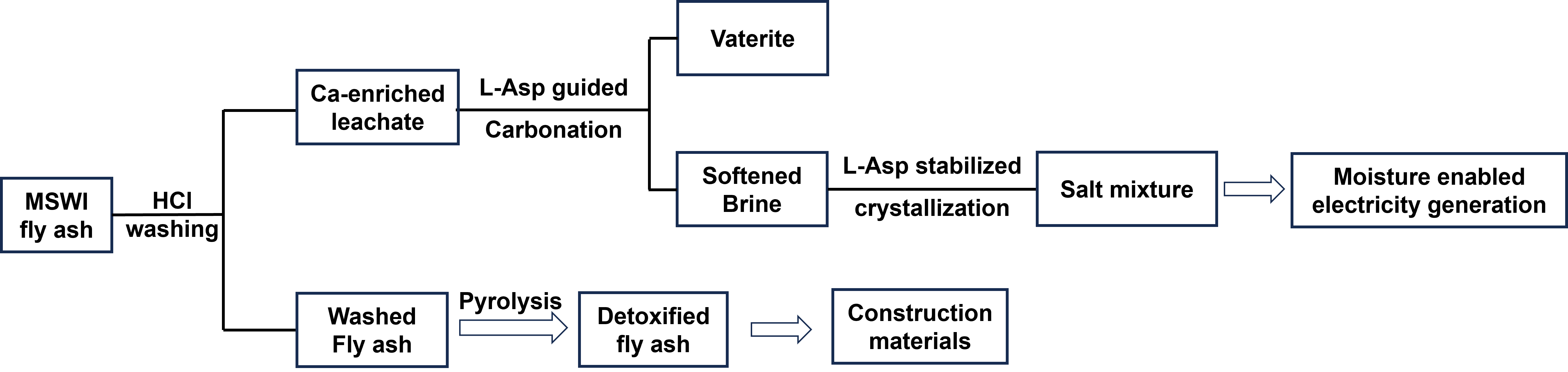


**Figure S19.** The technical route scheme of treating the Fly-ash.

**Table S1.** The detailed composition of MSWI fly ash gained by XRF.

| **Element** | **Content (%)** |
| --- | --- |
| Na | 1.58 |
| Mg | 0.04 |
| Al | 0.45 |
| Si | 1.27 |
| P | 0.39 |
| S | 3.94 |
| Cl | 37.32 |
| K | 9.13 |
| Ca | 42.13 |
| Cr | 0.02 |
| Mn | 0.04 |
| Fe | 1.24 |
| Cu | 0.12 |
| Zn | 1.12 |
| Br | 0.21 |
| Pb | 0.27 |

**Table S2.** Major cation concentration in mother liquor from sintering dust washing leachate (g/L)

| Rb | Cs | K | Na | Ca | Mg |
| --- | --- | --- | --- | --- | --- |
| 6.08 | 2.15 | 46.1 | 50.0 | 0.012 | - |

**Table S3.** National standard for leaching toxicities of MSWI fly ash (HJ 1134-2020)

| **Element** | **Control (ppm)** |
| --- | --- |
| Hg | 0.05 |
| Cu | 40 |
| Zn | 100 |
| Pb | 0.25 |
| Cd | 0.15 |
| Ba | 25 |
| Ni | 0.5 |
| Cr | 1.5 |

**Table S4.** XRF results of derived salts in acid pickling of MSWI fly ash leachate

| Number | Element | Content (%) | |
| --- | --- | --- | --- |
|  |  | Value | Error |
| 1 | Na | 20.65 | 2.07 |
| 2 | Mg | 0 | 0 |
| 3 | Al | 0 | 0 |
| 4 | Si | 0 | 0 |
| 5 | P | 0.57 | 0.01 |
| 6 | S | 0.45 | 0.16 |
| 7 | Cl | 66.52 | 4.34 |
| 8 | K | 11.01 | 2.43 |
| 9 | Ca | 0.55 | 0.05 |
| 15 | Br | 0.23 | 0.05 |
| 16 | Pb | 0 | 0 |

**Reference**

1. Kresse, G. and J. Furthmüller, *Efficiency of ab-initio total energy calculations for metals and semiconductors using a plane-wave basis set.* Computational materials science, 1996. **6**(1): p. 15-50.

2. Perdew, J.P., K. Burke, and M. Ernzerhof, *Generalized gradient approximation made simple.* Physical review letters, 1996. **77**(18): p. 3865.

3. Zhao, K., et al., *Single-ion chelation strategy for synthesis of monodisperse Pd nanoparticles anchored in MOF-808 for highly efficient hydrogenation and cascade reactions.* Nanoscale, 2022. **14**(30): p. 10980-10991.
